# Global analysis of translation termination in *E*. *coli*

**DOI:** 10.1371/journal.pgen.1006676

**Published:** 2017-03-16

**Authors:** Natalie E. Baggett, Yan Zhang, Carol A. Gross

**Affiliations:** 1 Department of Microbiology and Immunology, University of California, San Francisco, San Francisco, California, United States of America; 2 Department of Cell and Tissue Biology, University of California, San Francisco, San Francisco, California, United States of America; 3 California Institute of Quantitative Biology, University of California, San Francisco, San Francisco, California, United States of America; The Ohio State University, UNITED STATES

## Abstract

Terminating protein translation accurately and efficiently is critical for both protein fidelity and ribosome recycling for continued translation. The three bacterial release factors (RFs) play key roles: RF1 and 2 recognize stop codons and terminate translation; and RF3 promotes disassociation of bound release factors. Probing release factors mutations with reporter constructs containing programmed frameshifting sequences or premature stop codons had revealed a propensity for readthrough or frameshifting at these specific sites, but their effects on translation genome-wide have not been examined. We performed ribosome profiling on a set of isogenic strains with well-characterized release factor mutations to determine how they alter translation globally. Consistent with their known defects, strains with increasingly severe release factor defects exhibit increasingly severe accumulation of ribosomes over stop codons, indicative of an increased duration of the termination/release phase of translation. Release factor mutant strains also exhibit increased occupancy in the region following the stop codon at a significant number of genes. Our global analysis revealed that, as expected, translation termination is generally efficient and accurate, but that at a significant number of genes (≥ 50) the ribosome signature after the stop codon is suggestive of translation past the stop codon. Even native *E*. *coli* K-12 exhibits the ribosome signature suggestive of protein extension, especially at UGA codons, which rely exclusively on the reduced function RF2 variant of the K-12 strain for termination. Deletion of RF3 increases the severity of the defect. We unambiguously demonstrate readthrough and frameshifting protein extensions and their further accumulation in mutant strains for a few select cases. In addition to enhancing recoding, ribosome accumulation over stop codons disrupts attenuation control of biosynthetic operons, and may alter expression of some overlapping genes. Together, these functional alterations may either augment the protein repertoire or produce deleterious proteins.

## Author summary

Proteins are the cellular workhorses, performing essentially all of the functions required for cell and organismal survival. But, it takes a great deal of energy to make proteins, making it critical that proteins are made accurately and in the proper time frame. After a ribosome synthesizes a protein, release factors catalyze the accurate and timely release of the finished protein from the ribosome, a process called termination. Ribosomes are then recycled and start the next protein. We utilized ribosome profiling, a method that allows us to follow the position of every ribosome that is making a protein, to globally investigate and strengthen insights on termination fidelity for cells with and without mutant release factors. We find that as we decrease release factor function, the time to terminate/release a protein increases across the genome. We observe that the accuracy of terminating a protein at the correct place decreases on a global scale. Using this metric we identify genes with inherently low termination efficiency and confirm two novel events resulting in extended protein products. In addition we find that beyond disrupting accurate protein synthesis, release factor mutations can alter expression of genes involved in the production of key amino acids.

## Introduction

Ribosomes translate the genetic information in the mRNA to a linear sequence of amino acids in the polypeptide chain through a process consisting of initiation, elongation, termination and recycling. During initiation, the 30S subunit of the bacterial ribosome and various initiation factors assemble at the initiation codon on the mRNA. Elongation commences after the 50S subunit of the ribosome joins the complex. Cognate aminoacyl tRNAs, together with elongation factors decode the mRNA sequentially, binding first at the acceptor site (A site), followed by movement to the P site after amino acid transfer to the polypeptide chain at the peptidyl-transferase center. Several layers of error correction minimize the misincorporation of non-cognate amino acids [1,2]. Termination is signaled when a stop codon (UAA, UAG, UGA) enters the A site of the ribosome, where it is recognized either by release factor (RF) 1 or 2 [3]. RF1 or RF2 hydrolyze the polypeptide chain to terminate translation, and are then dissociated from the ribosome by RF3 during recycling [4]. Peptide release is a high fidelity process (error frequency of approximately 10^−5^), ensuring that stop codon recognition precedes peptide release [5–7]. Finally, the ribosome is dissociated to its 30S and 50S subunits by elongation factor EF-G, and ribosome recycling factor (RRF) [8–10].

There is increasing structural and biochemical understanding of the three bacterial release factors. RF1 and RF2 are structural mimics of an aminoacyl tRNA and both are essential in native *E*. *coli* K-12 [11,12]. They bind in the A site using conserved protein motifs in Domain 2 to recognize the 2^nd^ and 3^rd^ positions of the stop codons (RF1: UAA and UAG; RF2: UAA and UGA) [13]. Their universally conserved GGQ amino acid motif then reaches into the peptidyl transferase center to release the peptide chain by catalyzing its hydrolysis from the tRNA [12,13]. Methylation of RF1 and RF2 by PrmC at their GGQ motif enhances release factor activity [14].

Interestingly, *E*. *coli* K-12 strains have an RF2 variant with Thr at position 246 rather than the canonical Ala246 or Ser246 [15]. All other bacteria, including other *E*. *coli* lineages, have Ala or Ser at position 246 [16]. In this work, we call the *E*. *coli* K-12 RF2 variant, RF2^K-12^, and the *E*. *coli B* variant with Ala246, RF2^B^. RF2^K-12^ is discrepant from RF2^B^ in its properties. First, RF2^K-12^ has reduced ability to catalyze hydrolysis of the peptide bond to terminate translation relative to RF2^B^ [15]. Second, RF2^K-12^ but not RF2^B^ is almost completely dependent on methylation for activity [14]. Third, UAA decoding is done primarily by RF1 in strains with RF2^K-12^, but by both RF1 and RF2 in strains with RF2^B^ [14,15]. Indeed, RF1 is non-essential in an *E*. *coli* K-12 strain with the RF2^B^ variant [17,18]. Finally, because the level of RF2 is tuned to need via an internal UGA frameshifting event necessary to produce the full-length protein, RF2^K-12^ is present at a higher level than RF2^B^ as expected from its lower activity [14,19,20].

RF3, a non-essential release factor of *E*. *coli*, promotes dissociation of RF1 and RF2 from the ribosome [21,22]. This reaction occurs slowly in the absence of RF3 [4,23]. RF3 is a homologue of EF-G, a GTPase translocation factor that catalyzes movement of tRNA on the ribosome and ribosome dissociation [24]. The current idea is that RF3-GTP binds to the ribosome in the same location as EF-G, and similarly induces inter-subunit rotation, which creates a steric clash with the bound RFs, promoting dissociation of RF1 and RF2 [25,26].

The role of the release factors in translation termination fidelity has been explored *in vivo* by measuring by release of small artificial peptides or *in vitro* by measuring frameshifting or stop codon readthrough in a synthetic constructs containing a known frameshifting site or premature stop codon [16,27–32]. These assays showed that RF2^B^ and methylated RF1 and RF2 terminated translation better than RF2^K-12^, or unmethylated RF1 and RF2 [9,14,16,33–35]. Additionally, temperature sensitive mutations within RF1 and RF2 increase stop codon readthrough on *in vitro* constructs [29,31,32,36,37]. Suppressors of these temperature sensitive mutations mapped to the *prfC* (RF3) locus [38]. Further studies indicate that strains lacking RF3 have increased stop codon readthrough of *lacZ* reporter constructs and increased frameshifting over the known *prfB* (RF2) frameshifting sequence [9,33–35,39]. Overexpression of RF3 was shown to decrease frameshifting over the *prfB* frameshifting sequence [34,39]. These studies and RF3 overexpression studies indicated that cooperative interactions between RF1/2 and RF3 improved termination efficiency [38]. *In vitro* studies also implicate RF2 and RF3 in post-peptidyl transfer quality control (post PT-QC), a mechanism for selectively terminating translation of polypeptides that have misincorporated amino acids, and phenotypes suggestive of post PT-QC were found *in vitro* and *in vivo* [34,40], but were not reproduced in K-12 strains with RF2^B^ [35]. Taken together, the work thus far indicates how release factor mutations alter translation termination at specific reporter constructs or known frameshifting sites. However, the effects of these mutations on translation termination have not been studied on a global scale or at physiologically relevant native gene loci.

To elucidate the global effects of these mutations and observe how they perturb the translatome, we used ribosome profiling to examine the behavior of ribosomes at stop codons, and compare the extent of recoding and readthrough events genome-wide in native *E*. *coli* K-12, with that in K-12 RF2^B^ cells and in both strains lacking RF3. We also examined changes in protein expression globally among the strains. We find significant differences in the duration of termination/release dependent upon strain background, identify new recoding events and reveal the impact of altered termination on genes whose expression is regulated by transcription-translation coupling.

## Results

### Growth rates at various temperatures

**Fig 1:**
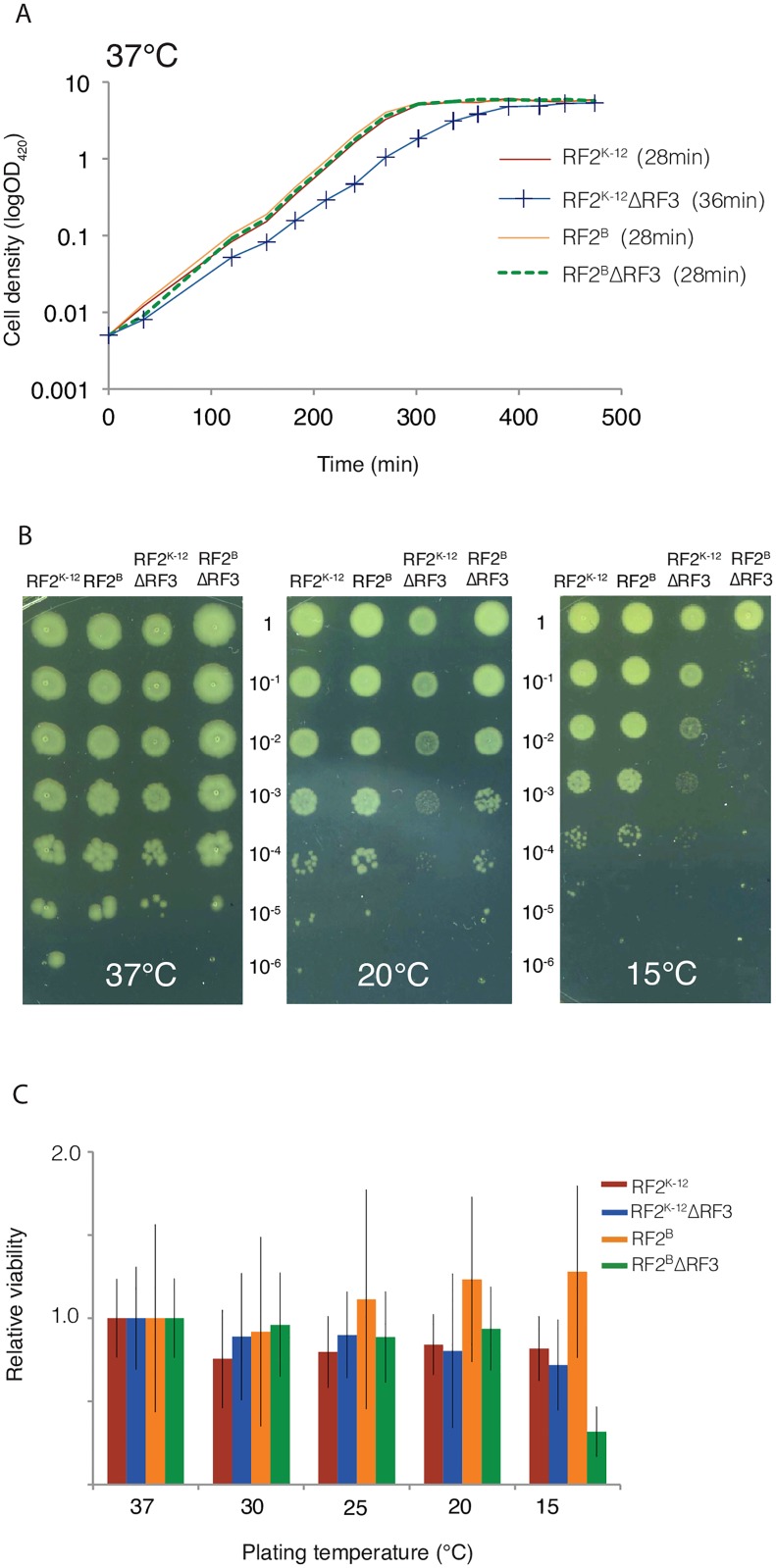
Growth defect and cold sensitivity rescue of RF mutants. (A) Growth curves of MG1655 K-12 RF2^K-12^, K-12 RF2^K-12^ΔRF3, K-12 RF2^B^, K-12 RF2^B^ΔRF3 growing in MOPS-complete glucose medium at 37°C. Doubling times are indicated. (B) Spot dilutions of cultures growing exponentially at 37°C in LB were spotted onto LB-agar plates and incubated at 37°C, 20°C, and 15°C. These indicate that the slow growth of K-12 RF2^K-12^ΔRF3 at 20°C is rescued in K-12 RF2^B^ΔRF3. (C) After growth to mid-exponential phase in liquid LB cultures serial dilutions of each culture were plated on LB-agar in triplicate. Colony forming units (CFUs) of each strain were calculated for each temperature and plotted relative to the CFU of that strain at 37°C. Error bars are the standard error calculated between replicates (See Methods).

We examined the effect of altered release factors on the growth of *E*. *coli* MG1655, the prototypical K-12 strain used in our studies. Previous results indicated inconsistent phenotypes for MG1655 and BW25113, another K-12 strain [34,35,41]. Here, we determine the growth rates of MG1655 (K-12 RF2^K-12^) and isogenic single and double mutant release factor derivatives (Table 1). At 37°C in MOPS-complete glucose medium supplemented with all amino acids, K-12 RF2^K-12^ ΔRF3 has a significantly slower growth rate than K-12 RF2^K-12^ (doubling times of 36 min and 28 min, respectively), but the slow growth phenotype of ΔRF3 is rescued by RF2^B^ (Fig 1A). This suggests that the enhanced activity of RF2^B^ compensates for the ΔRF3 defects that result in reduced growth rate. K-12 RF2^B^ and K-12 RF2^K-12^ have indistinguishable growth rates.

**Table 1:** *E*. *coli* strains and genotypes in this study.

Strain name	Genotype
**K-12 RF2**^**K-12**^ **(MG1655)**	F- λ- *ilvG- rfb-50 rph-1*
**K-12 RF2**^**K-12**^ **ΔRF3**	MG1655 *ΔprfC*::*frt*
**K-12 RF2**^**B**^	MG1655 *prfB* [*E*. *coli* B]
**K-12 RF2**^**B**^ **ΔRF3**	MG1655 *prfB* [*E*. *coli* B] *ΔprfC*::*frt*

Our previous global phenotyping screen of the *E*. *coli* BW25113 single gene deletion library indicated that ΔRF3 was quite cold-sensitive at 16°C and 20°C [42]. ΔRF3 strains in other backgrounds, both K-12 and non K-12 derived, also grow slowly at 25°C, a phenotype that is reversed by RF2^B^ΔRF3 [35]. We therefore characterized the growth rate of our isogenic *E*. *coli* K-12 MG1655 strains across an expanded temperature range. Consistent with previous results [35], K-12 RF2^K-12^ ΔRF3 exhibits severe cold sensitivity at 20°C, which was reversed in the K-12 RF2^B^ ΔRF3 strain (Fig 1B). Surprisingly, however, at 15°C, the RF2^B^ variant did not rescue ΔRF3 (Fig 1B). These results motivated us to examine strain viability at each temperature (Fig 1C). We find that although it is slow growing, K-12 RF2^K-12^ ΔRF3 maintains viability at all temperatures tested. In contrast, K-12 RF2^B^ ΔRF3 exhibited a near 5-fold decrease in viability at 15°C. Thus, at very low temperature the RF2^B^ variant is more deleterious than RF2^K-12^ variant when paired with RF3 deletion (see Discussion).

### Global analysis of translation termination/release in strains with altered termination efficiency

**Fig 2:**
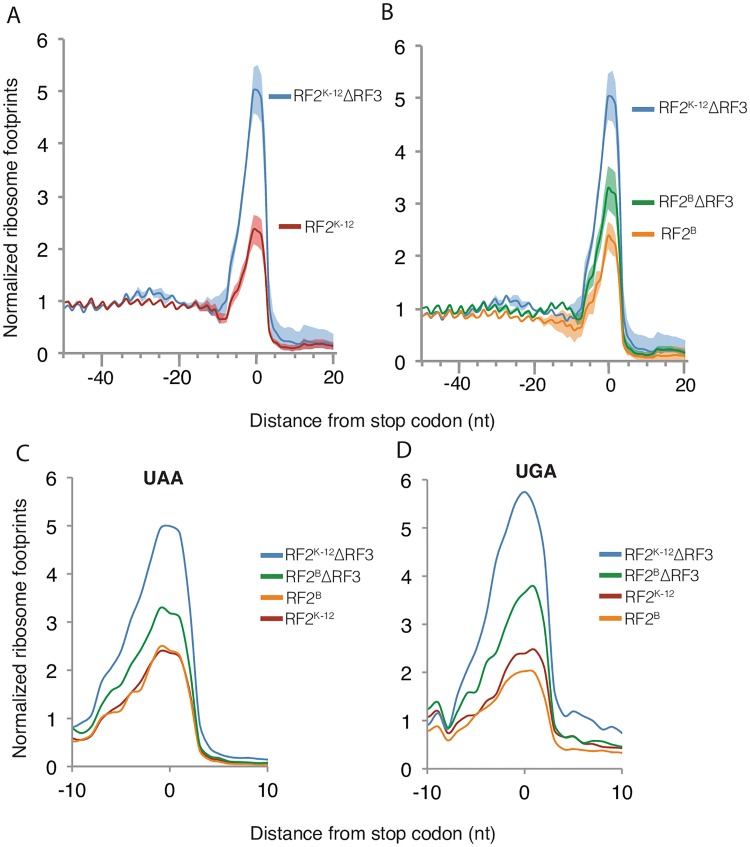
Ribosome occupancy over stop codons increases in the absence of RF3 in *E*.*coli* K-12. Metagene analysis of ribosome footprint density in the region surrounding stop codons. The approximately 1200 well-expressed genes were aligned at their stop codons and the median normalized ribosome density at each position was calculated from ribosome profiling data of strains grown in MOPS complete-glucose media at 37°C. (A and B) Median normalized density (solid line) with standard error (shaded region) across repeat experiments. (A) Ribosome stop codon density for K-12 RF2^K-12^ΔRF3 (2 replicates) versus K-12 RF2^K-12^ (4 replicates). (B) Ribosome stop codon density for K-12 RF2^B^ΔRF3 (5 replicates) versus K-12 RF2^B^ (2 replicates). (C and D) Average normalized density across replicates at UAA stops (947 genes) and UGA stops (231 genes).

We performed ribosome profiling for K-12 RF2^K-12^ and K-12 RF2^B^ strains, with and without RF3. All ribosome profiling experiments were performed at 37°C in MOPS- complete glucose medium because it contains a balanced complement of amino acids (see Methods). As a proxy for duration of ribosome termination and release, we quantified the extent of ribosome occupancy at stop codons. The density of footprints at any codon is related to the dwell time of the ribosome at that position [43]. Thus, higher stop codon occupancy is indicative of increased duration of termination/release. We note that this metric is a composite measurement that minimally consists of the rates of: release factor binding, polypeptide chain termination, release factor release, and ribosome recycling. Our initial analysis used a dataset of approximately 1200 well-expressed genes aligned at the stop codons of their open reading frames (ORFs), and is comprised of 947 UAA stop codons, recognized by both RF1 and RF2, and 231 UGA codons recognized only by RF2 (Fig 2, see Methods). For additional analyses, we expanded the dataset to 3390 genes so that we could also study the lower abundant UAG stop codons, as well as all of the 4-base stop codons (S1 Fig, see Methods).

We used our 1200 gene dataset to compare global stop-codon occupancy by native K-12 RF2^K-12^ and K-12 RF2^K-12^ ΔRF3 cells, as well as to compare K-12 RF2^B^ cells and K-12 RF2^B^ ΔRF3 cells. We found that K-12 RF2^K-12^ ΔRF3 cells have a global 2.5-fold increase in ribosome occupancy at stop codons relative to native K-12 RF2^K-12^, indicating a significant defect in termination/release (Fig 2A). K-12 RF2^B^ ΔRF3 cells also have a global increase in ribosome occupancy at stop codons relative to K-12 RF2^B^ (Fig 2B), but the magnitude of this increase is less (1.4-fold). It is likely that the more active RF2^B^ variant limits the effect of ΔRF3. Changes in occupancy are also evident in ribosome footprint density plots of single genes (S2 Fig).

Because global stop codon occupancy could be driven by the behavior at the highly abundant UAA stop codons, we next compared the behavior at each of the three stop codons. We first compared K-12 RF2^K-12^ΔRF3 and K-12 RF2^B^ ΔRF3 at all three stop codons. Changes in occupancy of the ΔRF3 strains at all three stop codons, including the low abundance UAG stop codon recognized only by RF1, mirrors their change in global occupancy (Fig 2C and 2D and S1B Fig). Occupancies over UAG are very similar to those over UAA, although the low number of UAG stops increases the noise in the data (S1B Fig). The defects of translation termination/release seen at all stop codons for strains lacking RF3 is consistent with previous studies showing that RF3 is important for release of both RF1 and RF2 [22]. Notably, relative to K-12 RF2^K-12^, the K-12 RF2^K-12^ΔRF3 strain has a very slight increase in ribosome occupancy ~20–30 bp upstream of the stop, at the position expected for a second ribosome (Fig 2A). The height of this shadow peak increases in highly translated genes (S3 Fig), suggestive of ribosome pileup. However, further experiments are necessary to definitively establish this point. In summary, deleting RF3 results in a general defect in termination/release at all three stop codons, but the magnitude of this effect is much smaller in K-12 RF2^B^ than in the native K-12 RF2^K-12^ strain.

We next compared the stop codon occupancy of K-12 RF2^K-12^ with that of K-12 RF2^B^ both globally (Fig 2A and 2B) and at specific stop codons (Fig 2C and 2D, S1B Fig). Both strains have similar stop codon occupancy globally, as well as at the UAA and UAG stop codons (Fig 2A–2C, S1B Fig). However, for UGA codons, recognized solely by RF2, there is a general trend towards decreased occupancy in K-12 RF2^B^ versus K-12 RF2^K-12^ strain, consistent with observations that the RF2^B^ protein is more efficient at mediating termination at UGA than RF2^K-12^ variant (Fig 2C and 2D, S1B Fig) [15,16].

The base following the stop codon, called the fourth base, is known to impact the efficiency of stop codon termination and is recognized by both RF1 and RF2 [39,44,45]. We created a metagene plot for each 4-base stop codon to examine the occupancy preferences of each of our strains. While these plots generally have more noise than the 3-base stop plots due to a smaller dataset for each 4-base stop codon, particularly on the lower abundant UAG and UGA stops, we can observe general trends of occupancy within each specific stop codon group (S1 Fig). Despite noise, behavior of strains at the 4-base codons generally mirrored their behavior at the 3-base codons, with some additional patterns. The K-12 RF2^B^ strain has the most severe decrease in stop codon occupancy relative to K-12 RF2^K-12^ at nearly all 4-base UGA stop codons (S1 Fig). Strikingly, at the UGAA stop, K-12 RF2^B^ ΔRF3 has ribosome occupancy equivalent to that of native K-12 RF2^K-12^, rather than the slightly elevated level characteristic at other stops. Together, these results suggest that RF2^B^ variant is significantly more effective than the RF2^K-12^ protein at UGAA stops (see Discussion).

### Release factor expression

**Fig 3:**
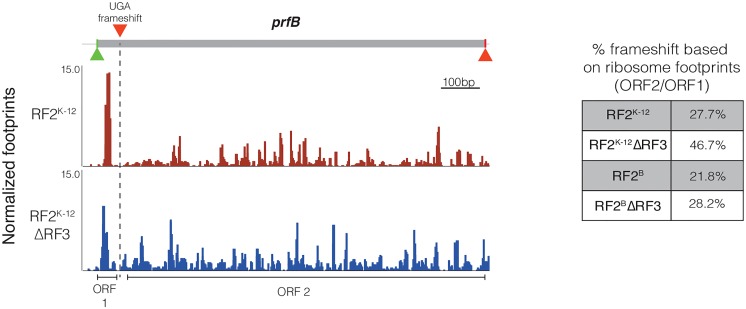
Increased rate of programmed frameshifting at the *prfB* locus. The translation of *prfB* is regulated through a programmed +1 frameshift at codon 26, shown as a UGA frameshift (dotted line). Successful frameshifting results in the complete RF2 protein. Ribosome profiling data was used to estimate frameshifting over the internal UGA stop codon by comparing the ribosome occupancy (RPKM) across the first small ORF prior to the UGA stop and second ORF encoding full-length RF2. This ratio gives a rough estimate of percent frameshift at the *prfB* locus for each RF mutant.

We also used the ribosome profiling data to examine release factor expression in each strain. Previous experiments using reporter constructs indicated that the rate of frameshifting at the internal *prfB* UGA stop codon used to change expression of RF2, increased in a ΔRF3 strain [34,39]. We show that this result also is true at the endogenous *prfB* locus by calculating the ratio of ribosome occupancy of the larger second frameshifted ORF (encoding full length RF2) relative to that of the smaller first ORF. Native K-12 RF2^K-12^ cells exhibit ~28% frameshifting at this locus and this value increased to ~47% in the K-12 RF2^K-12^ ΔRF3 strain (Fig 3). K-12 RF2^B^ cells appear to have slightly less frameshifting (21%) than K-12 RF2^K-12^ cells. K-12 RF2^B^ΔRF3 cells do not exhibit the increased frameshifting as seen in RF2^K-12^ΔRF3 cells, but instead exhibit a similar amount of frameshifting as K-12 RF2^K-12^.

Concomitantly, we measured the overall expression levels of RF2, finding general agreement with our calculated rate of frameshifting. K-12 RF2^K-12^ cells have a higher level of RF2 than K-12 RF2^B^ cells (Table 2), consistent with the previously reported ~40% reduction of RF2 in K-12 RF2^B^ calculated by quantitative western blot [14]. Additionally, as expected from increased frameshifting, K-12 RF2^K-12^ ΔRF3 cells have increased expression of RF2 relative to K-12 RF2^K-12^ cells (Table 2). K-12 RF2^K-12^ ΔRF3 cells also have increased expression of RF1 relative to K-12 RF2^K-12^ cells. The fact that K-12 RF2^K-12^ ΔRF3 cells exhibit severe termination/release defects despite increased release factor expression suggests that increased expression only partially mitigates this phenotype. Neither RF2 nor RF1 expression increases in K-12 RF2^B^ΔRF3 relative to K-12 RF2^B^, presumably because the higher activity of the RF2^B^ protein relative to RF2^K12^ prevents the signals triggering enhanced release factor expression.

**Table 2:** Expression of release factors in K-12 and release factor mutants. Gene expression as measured by ribosome footprint density (reads per kilobase of transcript per million mapped reads, RPKM) for the three release factors *prfA*, *prfB*, and *prfC* in MOPS-complete glucose medium at 37°C. The average ribosome density and standard error of the mean were calculated from replicate data sets. Gene expression is proportional to the rate of protein synthesis [43].

Locus	RF2^K-12^	RF2^K-12^ΔRF3	RF2^B^	RF2^B^ΔRF3
*prfA* (RF1)	82.8 ± 10.7	118.7 ± 15.0	87.1 ± 17.1	74.3 ± 14.8
*prfB* (RF2)	736.7 ± 113.7	1069.5 ± 77.7	526.2 ± 57.8	508.0 ± 99.3
*prfC* (RF3)	438.9 ± 80.7	--	461.7 ± 120.5	--

### ΔRF3 strains exhibit higher ribosome density in the post-ORF region

**Fig 4:**
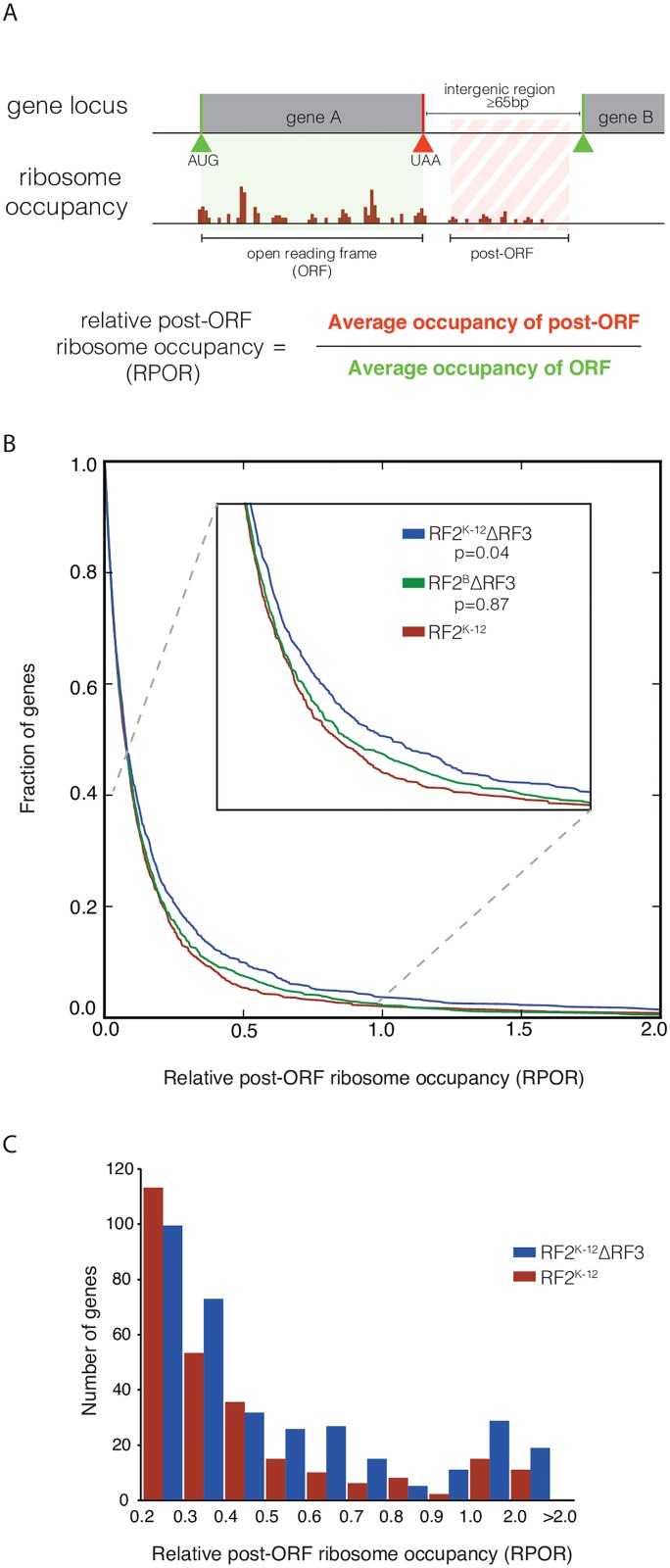
Genome wide increase in post-ORF occupancy in K-12 ΔRF3. (A) Ribosome occupancy in the region past the annotated stop codon was calculated for all genes that had an intergenic region of 65 bases or greater, measured from the stop codon of the upstream gene of interest to the start codon of the downstream gene, and met expression thresholds for both mRNA abundance and ribosome footprints (1656 genes for the deepest sequenced library). The metric relative post-ORF ribosome occupancy (RPOR) was calculated from the average occupancy over the annotated ORF and the average occupancy in a post-ORF window 20-60bp after the stop codon. By using the average occupancy for both the post-ORF and ORF, we reduce the impact of length in these calculations. (B) The distribution of RPOR values from 0 to 2.0 for K-12 RF2^K-12^, K-12 RF2^K-12^ΔRF3, and K-12 RF2^B^ΔRF3 is shown as a cumulative distribution function for one experiment with 1139 genes after all zero RPOR values were removed. A shift towards higher RPOR values in the K-12 RF2^K-12^ΔRF3 strain relative to the K-12 RF2^K-12^ strain is statistically significant (p-value 0.04; Kolmogorov-Smirnov (K-S) test), but the small shift between the K-12 RF2^B^ΔRF3 and K-12 RF2^K-12^ strains is not (p-value 0.84, K-S test). (C) A histogram of the distribution of RPOR values ≥0.2 for K-12 RF2^K-12^ and K-12 RF2^K-12^ΔRF3 compares the number of genes in each range of RPOR for both strains.

Extended stop codon occupancy could have the downstream consequence of facilitating either stop codon readthrough or of frameshifting, collectively called here recoding events. Our finding of increased programmed frameshifting at the *prfB* locus in certain strain backgrounds is consistent with that idea. Recoding events would result in an increase in ribosome density after the annotated stop codon. We therefore quantified the average ribosome density downstream of the open reading frame (ORF) relative to the average density within the ORF itself. We call this metric relative post-ORF ribosome occupancy (RPOR). We analyzed RPOR for the almost 1600 genes that are well separated from their immediate downstream genes, i.e. having ≥ 65 nucleotides between the stop codon of the upstream gene and the start codon of the downstream gene (Fig 4A). The distance constraint is necessary to enable us to unambiguously examine ribosomes past the stop codon of the gene without interference from translation of the downstream gene.

A plot of the cumulative distribution of RPOR values indicates that most genes have very low RPOR values across all strains. Indeed, nearly 60% of genes had an RPOR value under 0.1, indicative of very few ribosomes in the post-ORF region (Fig 4B). Thus, as expected, translation termination has high fidelity and is generally efficient [1,7]. However, K-12 RF2^K-12^ΔRF3 trends towards higher RPORs than K-12 RF2^K-12^ across the entire range of RPOR values (Fig 4B and S4A Fig), indicating a potential for globally reduced termination efficiency across the translatome. This shift is quite pronounced for those genes with the highest RPOR values: K-12 RF2^K-12^ΔRF3 has nearly twice as many genes with RPOR > 1.0 compared to K-12 RF2^K-12^ (Fig 4C). Although the RPOR values of K-12 RF2^B^ΔRF3 are not nearly as elevated as those of K-12^K-12^ΔRF3 (Fig 4B), replicate experiments suggest that they are elevated relative to the native K-12 RF2^K-12^ strain (S4B Fig). Finally, the RPOR values of K-12 RF2^B^ are very similar to those of K-12 RF2^K-12^. Our most complete K-12 RF2^B^ dataset showed a very slight decrease in RPOR values relative to K-12 RF2^K-12^ cells, but a smaller dataset did not exhibit this trend (S5 Fig).

**Fig 5:**
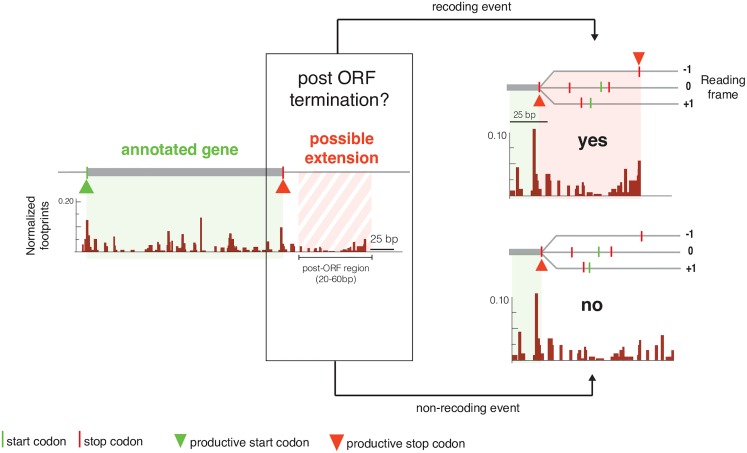
Schematic explaining ORF classification as recoding or non-recoding. Genes with high relative post-ORF ribosome occupancy (RPOR) were individually analyzed to determine the origin of ribosomes in the post-ORF region. Left hand panel: Schematic of ribosome footprints for an annotated gene (shaded light green) and its post-ORF region (shaded red stripes). The ORF start (green triangle) and stop (red triangle) codons are indicated. Right hand panel: a zoomed-in post-ORF region with all possible start (green line) and stop (red line) codons in each reading frame indicated. Upper right hand panel: A post-ORF region where ribosome density abruptly decreases after the stop codon in the -1 frame, classified as a putative recoding event. The hypothesized extended region is shaded in red and the putative stop codon is marked with a red triangle. Lower right hand panel: A post-ORF region where ribosome density does not decrease after any possible the stop codon, classified as a non-recoding event.

Ribosome profiling in other organisms, including *Saccharomyces cerevisae* and *Drosophila melanogaster*, is capable of producing protected ribosome fragments with reading frame information more precisely allowing for identification of recoding events [46,47]. However, bacterial ribosome footprints are generated with MNase, and reading frame information is lost because of the sequence specificities of this nuclease [48]. These specificities do not allow for perfect cutting of mRNA around the ribosome, resulting in variable ribosome footprint sizes, depending on sequence context, without reading frame information [49]. Therefore, we used a secondary criterion to identify those ORFs whose translation is likely extended into the post-ORF region as a result of readthrough or frameshifting. If the ribosomes found in the post-ORF region are actually translating, then translation should terminate after the ribosomes encounter a post-ORF stop codon in the translating reading frame. Therefore, we searched the data for ORFs exhibiting a reduction in ribosome density after a stop codon present in any frame of the post-ORF region relative to its density prior to that stop codon (Fig 5).

We hand-annotated the 100 genes with the highest relative post-ORF occupancy in both native K-12 RF2^K-12^ and K-12 RF2^K-12^ΔRF3 strains (121 total; S1–S4 Tables) to identify cases where there was a decrease in ribosome density coincident with a stop codon located in any of the three possible post-ORF reading frames. Using this criterion we classified 43 ORFs as likely recoding events (S1 Table). An additional 41 ORFs were classified as possible recoding events based on appropriate post-ORF termination with the addition of confounding factors such as low reads or other sequence elements that could contribute to high RPOR values (S2 Table). The vast majority of genes annotated as likely recoding events had a larger RPOR value in K-12 RF2^K-12^ΔRF3 than in native K-12 RF2^K-12^ cells (S1 Table). Interestingly, UGA codons were significantly over-represented (p-value = 1x10^-4^) in both likely and possible recoding events, occurring at nearly double their expected frequency, while UAA codons were de-enriched (S5 Table). We classified the 37 ORFs without reduced ribosome density after post-ORF stop codons as non-recoding events (S3 Table). We additionally found 4 cases of exceptionally high RPOR, which appear to stem from mis-annotations (*ydcM*, *yeaP*, *wbbK*, *yebW*) (S4 Table) and 12 ORFs that had too few reads to classify. Taken together, our data strongly suggest that native K-12 RF2^K-12^ has some recoding events and that deletion of RF3 in the K-12 background enhances recoding, likely due to poor termination/release efficiency.

### Genes with high post-ORF ribosome occupancy show extended protein products

**Fig 6:**
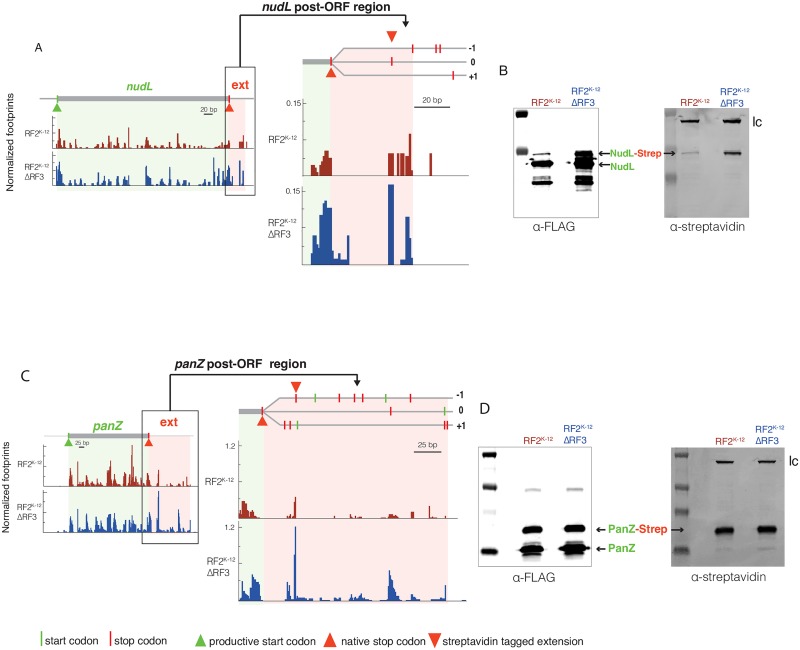
ΔRF3 increases recoding in genes identified with high ribosome occupancy post-ORF. (A and C) Left panels: Normalized ribosome footprints are shown across *nudL* (A) or *panZ* (C). The ORFs (shaded green) and post-ORF region, with hypothesized extensions (shaded red) are indicated. Right panels: zoomed-in post-ORF region with all possible start (green line) and stop (red line) codons in each reading frame indicated. The stop codon of the C-terminal streptavidin tagged extension is indicated with a red triangle within the shaded red extension. (B and D) Western blots of strains containing N-terminally FLAG-tagged and C-terminally streptavadin tagged NudL (B) or PanZ (D) and using SurA as a loading control (lc). The NudL C-terminal streptavidin tag is in the 0 frame, and that of PanZ is in the -1 frame. (A) The post-ORF region of *nudL* has reduction in ribosome occupancy correlated with stop codons in both the 0 and -1 frame. We determined the 0 frame produces an extended NudL product (S7 Fig). (B) Blotting of α-FLAG indicated the NudL protein, 22.78kDa, and the readthrough extension product at 24.06kDa which is also seen in the α-streptavidin blot. (C) The post-ORF region of panZ reveals several possibly productive stop codons. (D) Blotting of α-FLAG indicated the PanZ protein, 15.84kDa, and the -1 frame extension product at 20kDa, which is also seen in the α-streptavidin blot.

We tested whether our screening criterion, reduced ribosome density after a stop codon in the post-ORF region, identifies genes with a recoding event for three individual genes: *nudL* and *panZ* identified in this work, and *pheL*, a known frameshifter (S1 Table) [50]. We individually expressed the three genes on a multi-copy plasmid in all strain backgrounds; they all contained an N-terminus FLAG-tag, and the two newly identified putative recoding events were additionally tagged with streptavidin on the C-terminus of the suspected extension frames. We tested for protein extension products with quantitative Western blotting. This revealed that all three genes exhibited extended proteins in native K-12 RF2^K-12^, and that two have increased extended product in K-12 RF2^K-12^ΔRF3 (Fig 6B, S6 Fig).

The *nudL* post-ORF region ribosome density decreased significantly after two closely spaced stop codons, suggesting either readthrough (0 frame) or a -1 frameshift (Fig 6A). By constructing C-terminal streptavidin tags in both frames, we were able to show that the protein extension product resulted from stop codon readthrough in the 0 frame (S7 Fig). The extension product is approximately 6-fold more prevalent in K-12 RF2^K-12^ΔRF3 than in K-12 RF2^K-12^ (Fig 6B) and is undetectable in strains with RF2^B^ even when they lack RF3 (S8A Fig).

Ribosome density in the post-ORF region of *panZ* is complex with many possible points of termination (Fig 6C). We tested whether there was extension in the -1 frame, as predicted by the major decrease in ribosome density after the first stop codon in the post-ORF region. We observe a large amount of the -1 frameshift product in all strains, accounting for ~35% of the total PanZ production (Fig 6D, S8B Fig). The high level of PanZ extension product may result from unknown *cis* element(s) that leads to frameshifting.

The three well-established frameshifting events in native K-12 *E*. *coli* are RF2 (encoded by *prfB*), PheL, and DnaX [51]. We discussed the frameshift used to control the amount of full-length RF2 above (Fig 3). Here we describe our studies of PheL, the other established locus where a frameshift event produces an extended product. Our ribosome profiling data showed decreased ribosome density after a stop in the 0 frame (S6A Fig), in addition to the previously identified +1 frameshift, which ends in the downstream *pheA* gene [52]. Using N-terminal FLAG-tagged PheL, we see both products in K-12 RF2^K-12^, with enhanced production in K-12 RF2^K-12^ ΔRF3 cells (S6B Fig). However, we find no extended FLAG-PheL products in K-12 RF2^B^ or K-12 RF2^B^ΔRF3 cells (S6B Fig). Interestingly, native K-12 RF2^K-12^ cells have an unusually large ribosome density over the *pheL* UGAA four-base stop codon (S6A Fig). This enhanced density is almost completely eliminated in both K-12 RF2^B^ and K-12 RF2^B^ΔRF3 cells (S6A Fig), likely because UGAA signals rapid termination in cells with RF2^B^ protein variant.

### Coupled genes in polycistronic operons

Genes in *E*. *coli* are densely packed in the chromosome, with approximately 15% of adjacent ORF pairs having overlapping stop and start codons [53]. In some cases, this overlap has been shown to promote translational coupling, possibly by enabling upstream ribosomes to influence downstream ORF translation by unwinding mRNA structure or affecting ribosome dissociation-reinitiation cycle [54–58]. We asked whether the relative translation level of downstream genes (normalized to that of upstream genes) increased in K-12 RF2^K-12^ΔRF3 as compared to native K-12 RF2^K-12^ for adjacent ORF pairs with overlapping stop and start codons. We find that when the upstream gene has higher translation than the downstream gene in native K-12 RF2^K-12^ cells, deleting RF3 can increase translation of the downstream gene (S9A Fig). The aberrantly high translation was rescued in K-12 RF2^B^ ΔRF3, and K-12 RF2^B^ is similar to native K-12 RF2^K-12^ (S9B and S9C Fig). Accumulation of ribosomes at the upstream stop codon may promote unwinding of the mRNA structure at the translation initiation region of the downstream gene to increase its translation level, or alternatively, may be an example of readthrough.

### ΔRF3 increases attenuation of biosynthetic genes under control of leader peptide

**Fig 7:**
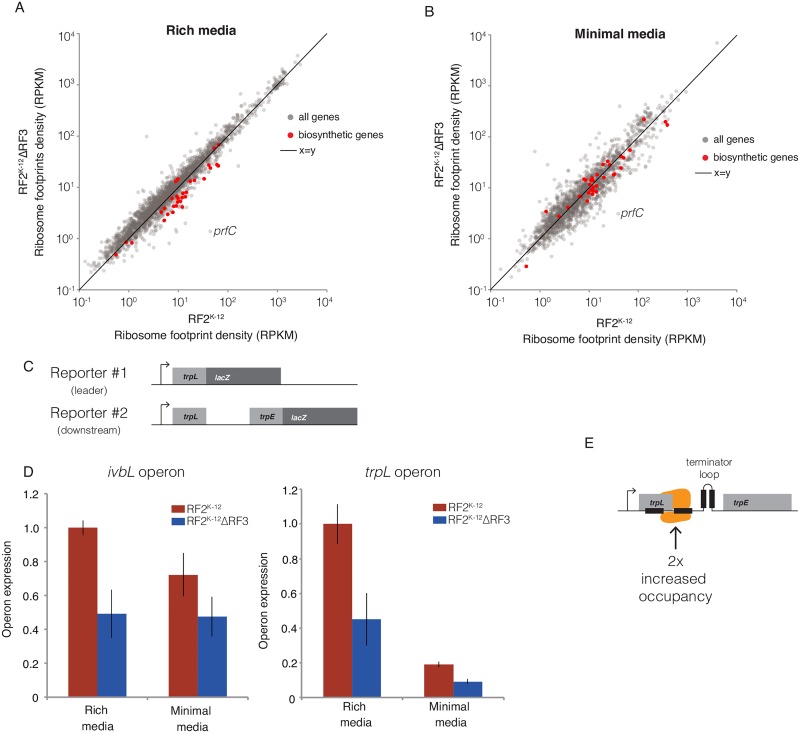
ΔRF3 reduces expression of biosynthetic genes controlled by leader peptide attenuation. (A and B) Scatter plots of ribosome footprint density in RPKM for K-12 RF2^K-12^ and K-12 RF2^K-12^ΔRF3 strains for all genes above minimum read threshold, with biosynthetic genes under the control of leader peptide attenuation in red are all others in grey. (A) Rich media samples of K-12 RF2^K-12^ and K-12 RF2^K-12^ΔRF3 grown in MOPS-complete glucose medium are averaged data across multiple replicates. (B) Minimal medium ribosome footprint densities between K-12 RF2^K-12^ and K-12 RF2^K-12^ΔRF3 in MOPS minimal glucose medium are plotted for a single replicate. (C) Schematic of the chromosomally integrated reporter constructs measuring expression of the leader peptide (reporter #1) or downstream gene expression (reporter #2), both under control of their native promoter. (D) Operon expression in K-12 RF2^K-12^ and K-12 RF2^K-12^ΔRF3 strains was calculated by dividing the β-galactosidase activity of reporter #2 by activity of reporter #1 and normalizing to the K-12 RF2^K-12^ strain in in MOPS complete-glucose rich media. (E) Increased attenuation in K-12 RF2^K-12^ΔRF3 strain may result from increased occupancy over the stop codon and post-ORF region. This mechanism would stabilize the formation of the downstream terminator loop over the anti-terminator loop.

Expression of many *E*. *coli* biosynthetic genes is controlled by regulated transcription termination, or attenuation. Attenuation is mediated by two competing RNA stem-loop structures, one signaling transcription termination and the other allowing transcription to continue [59]. Ribosome occupancy in the leader peptide mRNA determines the ratio of the two stem-loop structures. Enhanced ribosome occupancy in the leader peptide, indicative of a deficiency of the amino acid produced by the biosynthetic operon, leads to enhanced transcriptional readthrough, thus ensuring adequate production of the limiting amino acid. We asked whether altered ribosome occupancy at leader peptide stop codons in the K-12 RF2^K-12^ΔRF3 strain altered attenuation. A comparison of ribosome footprint density of native K-12 RF2^K-12^ with that of K-12 RF2^K-12^ΔRF3 indicated that biosynthetic genes under the control of a leader peptide appeared to be down-regulated in K-12 RF2^K-12^ΔRF3 relative to native K-12 RF2^K-12^ in rich media (Fig 7A) and less so, if at all, in minimal media (Fig 7B).

We quantified the attenuation of two leader peptide operons, *ivbL* and *trpL*, in both MOPS-complete glucose, a rich medium, and MOPS-glucose, a minimal medium, using two chromosomally integrated *lacZ* reporters for each operon. Reporter #1 measured expression of the leader peptide to quantify the rate of transcription of the operon; and reporter #2 measured expression of downstream genes (Fig 7C). Relative to native K-12 RF2^K-12^, K-12 RF2^K-12^ ΔRF3 cells have reduced expression of *trp* genes in both rich and minimal medium, while *ivbL* has reduced expression in rich medium (Fig 7D). As reporter #1 allows us to normalize for rate of transcription, we conclude that this decrease in expression is the result of increased attenuation due to increased ribosome occupancy over the stop codon in K-12 RF2^K-12^ ΔRF3 cells (Fig 7E). We also performed these experiments for the *hisGDCBHAFI* operon but were unable to measure transcription, as reporter #1 was toxic. Results with reporter #2 indicated that expression of *his* was decreased in K-12 RF2^K-12^ ΔRF3 cells in rich but not minimal medium (S10 Fig). We believe that this also represents attenuation because expression of the *ivlBN* and *trpEDCBA* leader peptides were comparable in K-12 RF2^K-12^ and RF2^K-12^ΔRF3 strains, suggesting that the lack of RF3 may not alter transcription from the leader peptide promoter.

The results of expression data from our ribosome profiling experiments, coupled with confirmatory *lacZ* fusion experiments indicate that altered ribosome density over the stop codon alters the outcome of the RNA structure competition, such that cells with enhanced stop codon occupancy have increased transcription termination. Most likely, increased occupancy over the stop codon shifts the equilibrium between the readthrough and termination stem-loop structures to one favoring the termination stem-loop. These findings are consistent with and extend an early study showing that some temperature sensitive RF2 strains increased transcription termination in the *trp* operon [60]. In that study, enhanced termination was observed only at UGA stops, but in our study, possibly because of a stronger perturbation, we observe increased termination also at the UAG stop in the *ivbL* locus. These results indicate how the defects of release factor mutations can alter downstream gene expression depending on transcription-translation coupling.

## Discussion

It is critically important for organisms to terminate the translation of proteins accurately and in an appropriate time frame. Release factors are central to this process. In this work, we compared the accuracy of translation termination and its downstream consequences at a global scale in native *E*. *coli* K-12 cells, which have a reduced function RF2 protein (RF2^K-12^) with K-12 cells harboring a fully functional RF2 (RF2^B^), and examined the consequences of the deletion of RF3 in both strain backgrounds. Our results provide the first picture of the genome wide consequences of stressing translation on ribosome behavior and release factor synthesis in a panel of isogenic strains with increasingly deficient release factor activity.

We used ribosome occupancy over stop codons as our *in vivo* composite metric for translation termination and ribosome release. In native K-12 RF2^K-12^ cells, the average occupancy of ribosomes at stop codons is about 2-fold higher than that in the coding regions, reflecting the time required from stop codon recognition to ribosome release (Fig 2A). The faster rate of peptide hydrolysis by the RF2^B^ variant relative to RF2^K-12^ [16] is manifest as a reduction in ribosome stop codon occupancy in K-12 RF2^B^ at UGA stop codons, which are fully dependent on RF2 for polypeptide release (Fig 2D). This effect is particularly strong at the UGAA codon which has relatively weak binding to RF2 (S1F Fig) [39,44,61]. Weak association of RF2 with a stop codon may allow immediate dissociation of RF2 following hydrolysis, which is especially rapid in strains containing the more active RF2^B^ variant, thus further decreasing the time to ribosome dissociation. On the other hand, both K-12 RF2^B^ and K12 RF2^K-12^ strains have similar stop codon occupancies at UAA codons. UAA is decoded by both RF1 and RF2, with previous studies indicating that UAA is predominantly recognized by RF1 in strains with RF2^K-12^, but that RF2 plays a major role in recognizing UAA codons in strains with RF2^B^ [14,15,30]. Thus, the higher activity of RF2^B^ as compared to RF2^K-12^ is manifest only at the UGA codon, recognized solely by RF2.

Deletion of RF3 globally perturbs stop codon occupancy by ribosomes, both for K-12 RF2^B^ and K-12 RF2^K-12^ strains. K-12 RF2^B^ ΔRF3 exhibits about a 1.4-fold increase in occupancy at all stop codons relative to K-12 RF2^B^ (Fig 2B–2D), indicating that the absence of RF3-mediated release factor dissociation visibly increases the dwell time of ribosomes at stop codons. Because of the composite nature of our measurement, we cannot say whether the effect we see is commensurate with the expectation from *in vitro* studies that loss of RF3 decreases the rate of release factor dissociation by as much as 500-fold or whether our results indicate that EF-G and RRF may partially compensate for RF3, as has been suggested [4,62,63]. For K-12 RF2^K-12^ ΔRF3 cells, the global perturbation of stop codon occupancy by ribosomes is more severe, exhibiting an almost 2.5-fold increase in ribosome occupancy at all stop codons relative to native K-12 cells (Fig 2A), a 1.5-fold increase in expression of both RF1 and RF2 (Table 2), and ≥20% increase in doubling time (Fig 1A). As the level of RF2 is adjusted by an internal frameshift, the rate of frameshifting changes in concert with the change in protein levels (Fig 3).

RF1 and RF2 are sub-stoichiometric with respect to ribosomes: RF1 is 100-fold less abundant; and RF2 is 12-fold less abundant than the number of ribosomes in K-12 RF2^K-12^ cells [37,43,64]. This level of release factors is normally sufficient for efficient termination because release factors act catalytically on the small subpopulation of terminating ribosomes, and are then rapidly released from the ribosome by RF3 action [9,33]. However, in the absence of RF3, the much slower dissociation of RFs increases the dwell time of RFs at stop codons, which in turn leads to a decrease in the cellular concentration of free RFs. This effect is exacerbated when it is coupled with the defective peptide release RF2^K-12^ protein, leading to even longer dwell time of release factors and further depletion of pool of free RFs. The cell responds by increasing expression of RF1 and RF2 in the K-12 RF2^K-12^ΔRF3 strain, but this is insufficient to counteract sequestration of RFs as termination is globally slowed at all stop codons, including UAG, which is recognized solely by RF1 (Fig 2, S1 Fig).

Our studies stressed translation in K-12 RF2^K-12^ by removing RF3. Previous studies indicated that perturbing translation in K-12 RF2^K-12^ by inactivating the pseudouridine synthase, *rluD*, or the RF methyltransferase, *prmC*, were ameliorated by the RF2^B^ variant [14,65]. In K-12 *E*. *coli* deletion of these modification enzymes leads to a very severe growth defect, which is lethal in the case of K-12 Δ*prmC*. Therefore the relatively gentler perturbation by ΔRF3 in K-12 allows us to identify these changes in ribosomal occupancy that would be difficult or impossible to identify in K-12 deletions of *rluD* or *prmC*. Deletions of *prmC* or *rluD* are viable in backgrounds containing RF2^B^ and they would likely exhibit the similar molecular signature as the K-12 RF2^K-12^ΔRF3 strain, enhanced expression of RF1 and 2 coupled with a significant enhancement in ribosome occupancy at stop codons.

Two lines of evidence suggest that the enhanced ribosome occupancy seen at stop codons in cells with reduced RF2 function (e.g. RF2^K-12^) and lacking RF3 leads to translation readthrough and/or frameshifting at a significant number of genes. First, for 121 ORFs with the highest post-ORF ribosome occupancy, ≥30% of the genes have a decrease in ribosomal density after a stop codon in the post-ORF region (S1 Table). This signature is indicative of bona fide translation termination at those stop codons. Second, we rigorously confirmed recoding events in three cases. For the 2 new cases, we visualized the extension products by performing Western blotting on proteins that had distinct tags at both their N-terminus and at the C-terminus of the putative protein extension (Fig 6 and S6 Fig). These experiments reveal that 2 of the 3, *nudL* and *pheL* show readthrough of the UGA stop codon, notable because in bacteria readthrough events are rare relative to frameshifting events [51]. We favor the idea that near-cognate tRNAs for cysteine and tryptophan known to decode UGA stop codons [66,67] account for readthrough, as the sequence element required for using selenocysteine to decode UGA [68–70] is not present at these loci. Interestingly, PheL exhibits the previously documented +1 frameshift [52], in addition to the stop codon readthrough indicating that during their extended occupancy over the stop codon, both readthrough and frameshifting events are possible.

Programmed recoding can expand the repertoire of gene products and is utilized for gene regulation in bacteria, viruses, yeast and higher eukaryotes [51,71]. While most of the recoding previously found in *E*. *coli* produces functional proteins, as is the case for PrfB and DnaX, a functional role for +1 PheL frameshift seen in K-12 *E*. *coli* has not been found [52]. We believe that the bulk of the suspected recoding we found in *E*. *coli* simply reflects the limitations of the reduced function RF2^K-12^ protein, which terminates poorly at UGA stops relative to RF2^B^, a phenotype that is exacerbated by the absence of RF3. This idea is consistent with over-representation of UGA codons amongst ORFs likely to exhibit the greatest amount of recoding and with the strain specific behavior of the readthrough products of NudL and PheL and +1 frameshift product of PheL (S6 Table, S6 and S8 Figs). The NudL and PheL extended products increase about 5-fold in K-12 RF2^K-12^ΔRF3 relative to K-12 RF2^K-12^ but were not present in K-12 RF2^B^. Remarkably, they are also absent in K-12 RF2^B^ΔRF3. In these two cases, ΔRF3 alone may not be sufficient to drive recoding and might require a second perturbation coming from the weaker RF2^K-12^ variant. It is likely that many of the other UGA stop codons with very high post-ORF ribosome occupancy will show the same patterns as NudL and PheL. While we examined recoding only for ORFs with very high post-ORF ribosome occupancy, the shift towards higher occupancy in K-12 RF2^K-12^ΔRF3 strains occurs in majority of the 1600 genes examined (Fig 4B), potentially producing defective protein products, albeit at a very low level.

Why might the defects in translation termination of ΔRF3 in a K-12 background lead to increased recoding? As a consequence of release factor sequestration, the effective concentration of free RF1/2 is reduced, reducing termination efficiency and increasing the likelihood of recoding. During the increased time spend at stop codons, conformational flexibility in the complex may allow transient slippage and/or partial binding of an amino-acyl tRNA, which would be followed by binding of EF-G and subsequent elongation. The chances for EF-G binding are increased when cells lack RF3 because both proteins bind to the same position on the ribosome [13]. Recent studies of the kinetics of intragenic frameshifting reveal that it is accompanied by slower EF-G catalyzed ribosome translocation and a change in the fluctuating conformational states [72–74]. Because of the altered ribosome conformation at the stop codon in the absence of RF3, we speculate that such conditions may also occur during slippage at the stop codon, with its frequency determined by sequences surrounding the stop codon.

Given the sensitivity of *E*. *coli* K-12 to perturbations in translation termination, we wonder why this lineage does not have the more conserved, fully functional RF2^B^ protein. There are several possibilities. First, the alterations in gene expression for K-12 RF2^K-12^ relative to K-12 RF2^B^, in the presence or absence of RF3, may in aggregate be beneficial to the host, resulting in maintenance of the allele. Second, the lab strains derived from *E*. *coli* K-12 may not been under strong enough selective pressure to select for enhanced function release factor mutants. In support of this idea, when *E*. *coli* K-12 RF2^K-12^ is exposed to extreme translational stress by deleting Δ*rluD* (rRNA pseudouridine synthase), it acquires suppressors at the RF2 locus [65]. Finally, K-12 RF2^K-12^ may have acquired a compensatory mutation(s) enabling the reduced function allele to perform relatively well. Similar compensatory mutations have been documented in studies of antibiotic resistance [75]. If so, one might imagine that the compensatory mutations might be incompatible with the fully functional allele in some circumstances. The loss of viability of K-12 RF2^B^ ΔRF3 but not K-12 RF2^K-12^ ΔRF3 at very low temperatures is consistent with possibility that contemporary K-12 strains have acquired compensatory mutations, which increase their fitness.

## Materials and methods

### Bacterial strains and their construction

All experiments were performed in *E*. *coli* K-12 strain MG1655 or its derivatives. Our strain name and genotype of each strain in K-12 *E*. *coli* are as follows; K-12 RF2^K-12^: MG1655 *prfB* [*E*. *coli* K-12], K-12 RF2^K-12^ΔRF3: MG1655 *prfB* [*E*. *coli* K-12] *ΔprfC*::*frt*, K-12 RF2^B^: MG1655 *prfB* [*E*. *coli* B], K-12 RF2^B^ΔRF3: MG1655 *prfB* [*E*. *coli* B] *ΔprfC*::*frt*. The K-12 RF2^K-12^ΔRF3 strain was constructed by transducing the *prfC*::*kanR* locus from the KEIO collection into MG1655 and its *kanR* cassette was flipped out using pCP20 [76]. The K-12 RF2^B^ strain was constructed by transducing the *prfB* (RF2^B^) locus with *kanR* downstream into MG1655, The original *prfB* [*E*. *coli* B]: *kanR* construct was a gift of Lei Wang at The Salk Institute for Biological Studies. The linked *kanR* marker was removed by λ Red assisted homologous recombination to obtain K-12 RF2^B^ without markers [77]. K-12 RF2^B^ΔRF3 was constructed using standard P1 transduction of the KEIO locus into the K-12 RF2^B^ background.

### Growth experiments

Cultures for growth curve experiments were grown in rich defined liquid medium, MOPS-complete glucose overnight at 37°C then diluted to OD_420_ 0.005 in 35mL of fresh MOPS-complete glucose medium. Cultures were grown in a shaking water-bath incubator at 37°C with cell density measured by optical density at 420nm wavelength every 30 minutes until stationary phase. Cell densities over OD_420_ 0.5 were diluted to ensure we obtained values within the linear range of the spectrophotometer.

### Cold sensitivity experiments

The spotting assay of cold sensitivity was performed by culturing cells to mid-exponential growth in LB medium at 37°C and making serial 1 in 10 dilutions up to 10^−6^. We spot plated 3uL of each dilution onto LB plates in duplicate and incubated at 37°C, 30°C, 25°C, 20°C, and 15°C. Plates were removed from the incubators and imaged when individual colonies were visible. Cells for the cold viability experiments were prepared in the same manner and dilution series were plated on LB plates by glass beads in triplicate. Plates were incubated in the same temperature series and viable cells were counted once colonies were visible.

### Ribosome profiling

Ribosome profiling was performed as previously described by Oh et al [49]. All strains; K-12 RF2^K-12^, K-12 RF2^K-12^ΔRF3, K-12 RF2^B^ and K-12 RF2^B^ΔRF3 were grown at 37°C in MOPS-complete glucose, a rich defined media, as a liquid culture [78]. Previous ribosome profiling experiments have shown that ribosome pausing increases over serine codons during exponential growth in Luria broth due to serine being the first catabolized amino after sugar is utilized [78]. Use of MOPS-complete glucose eliminates these pauses and provides a more stable growth medium for *E*. *coli* during our experiments. Profiling was also performed in K-12 RF2^K-12^ and K-12 RF2^K-12^ΔRF3 strains in MOPS-glucose, a minimal defined media, for the results seen in Fig 7B. Cells from each strain were rapidly harvested by filtration and lysate was produced by pulverization of liquid nitrogen cooled samples. From this lysate ribosome footprints were created using MNase and total RNA was extracted for a simultaneous RNA-seq library production. Ribosome footprints between 24-32nt were isolated from the initial polyacrylamide sizing gel. Total RNA purified samples for RNA-seq were fragmented by alkaline fragmentation and 20-35nt fragments were isolated from the sizing gel. After ligation to Linker-1 the fragments were converted to a cDNA library and subsequent library preparations were performed as previously described [46,49]. Libraries were sequenced on an Illuminia HiSeq 2000 or HiSeq4000. Raw and processed data are available on the NCBI Gene Expression Omnibus (GEO) under the accession number GSE88725.

### Sequencing analysis

Generated sequencing reads were analyzed as previously described [46,49]. Reads were initially aligned to a genome file containing *E*. *coli* rRNA and tRNA sequences to computationally subtract rRNA and tRNA reads. Then the remaining unaligned reads were aligned to the *E*. *coli* genome NC_00913.2, reads with more than two mismatches were excluded as were reads aligning to multiple portions of the genome. Aligned reads between 20bp and 40bp in length were trimmed by 10bp on each side and center mapped on the genome as previously described [49]. Wiggle files were generated using the same read lengths and center mapping as described above and counts were normalized per million reads and visualized in IGV. To analyze post-ORF occupancies as outlined in Fig 4, we also aligned ribosome profiling reads to the 3’ end in accordance with a recent publication to increase reading frame information [79]. We found both center alignment and 3’ alignments of fragments led to the same conclusion. Gene expression for each gene was calculated in Plastid, a Python library for deep sequencing genomics, by masking occupancy at the first and last 5 codons within the annotated ORF, and normalized for read-depth and gene length by using RPKM (reads per kilobase of transcript per million mapped reads) [80]. Expression between two strains was only compared when genes each had at least 100 counts. Metagene analysis was performed by normalizing 70-100nt upstream of the stop codon within each gene then plotting the median for each position. Genes with less than 1 counts in this window and genes less than 50nt away from the downstream coding frame were excluded from the metagene analysis in Fig 2 (note: S1 Fig differs slightly with a lack of read count filter to include more genes). Signals from biological replicates of different samples were averaged and standard deviations were calculated respectively.

### Post-ORF region calculations

Genome wide calculations of ribosomes in the post-ORF occupancy were performed for all genes with an intergenic region of 65bp or greater that had average mRNA and ribosome footprint densities of 0.1 or greater (overlapping genes were excluded).

Post-ORF occupancy was determined by calculating the average density in the window 20-60bp downstream of the annotated stop codon. The start point of 20bp was chosen in order to eliminate the impact of ribosomes over the stop codon contributing to post-ORF counts. This window was utilized for all genes regardless if possible extensions were shorter or longer than that window. The average post-ORF occupancy of a gene was divided by the average occupancy of gene, which was calculated as described above, to obtain the relative post-ORF ribosome occupancy metric (RPOR). The RPOR roughly estimates the fraction of recoding occurring at each gene. For the cumulative distribution analysis all RPOR values of 0.0 were removed because we are unable to resolve between zero recoding events and insufficient read depth, the presence of zeros also falsely inflated the K-S statistic.

### Extended-ORF tagging and western blot

The pFLAG-MAC plasmid background was used to construct inducible N-terminal-FLAG, C-terminal streptavidin tagged plasmids for *nudL* and *panZ* while only the N-terminal-FLAG was used for *pheL*. We cloned in the gene locus from directly after the start codon extending past the annotated stop codon using restriction digest cloning with HindIII and EcoRI. Utilizing our ribosome profiling data we were able to identify the possible extension and cloned in a sufficient segment of each 3’-UTR, using predicted stop codons to place the C-terminal streptavidin tag. Each plasmid sequence was confirmed by Sanger sequencing and TSS transformed into K-12 RF2^K-12^, K-12 RF2^K-12^ΔRF3, K-12 RF2^B^ and K-12 RF2^B^ ΔRF3 strains. Strains containing our pFLAG-MAC constructs were cultured to mid-exponential growth and induced with 1mM IPTG for 2 hours and total cell protein was harvested using TCA precipitation. NudL and PanZ proteins were resolved on 12% Bis-Tris gels with MOPS running buffer. PheL protein was resolved on 16% Tricine-SDS gels with Tricine-SDS running buffer. Proteins were transferred onto nitrocellulose membranes via wet transfer then blocked using Li-Cor Odyssey PBS blocking buffer and incubated with primary antibodies α-FLAG (Sigma-F3165) and α-SurA at 1:5000 dilutions and α-streptavidin (abcam, ab76950) at a 1:2500 dilution overnight at 4°C. Membranes were washed with 1x PBS before incubation with Li-Cor α-mouse and α-rabbit secondary antibodies at 1:10000 dilutions and again washed before imaging. Membranes were imaged on a Li-Cor infrared imager. The 700nm and 800nm channels of the Li-Cor image allowed us to visualized both FLAG and streptavidin on the same membrane. Quantifications were performed using Image-J software and SurA as a loading control.

### Attenuation reporters and assay

Two reporter constructs were assembled for each biosynthetic locus; *hisL*, *trpL* and *ivbL* using an *attλ* integratable plasmid. The leader peptide reporter (#1) was constructed by cloning in the entire 5’UTR and leader peptide without its stop codon upstream of *lacZ* creating a translational fusion. The downstream reporter (#2) was constructed by cloning the entire 5’UTR, leader peptide, intergenic region and a small segment of the next gene downstream into the same plasmid backbone upstream of *lacZ*. Once all six plasmids were generated and validated by Sanger sequencing, we integrated them into both K-12 and K-12 RF2^K-12^ ΔRF3 cells. Integration was performed using CRIM helper plasmid pINT-ts and single integrants were confirmed by diagnostic PCR [81]. After multiple attempts we found the *hisL* leader peptide reporter to be toxic to the cell, likely due to the high production of the *lacZ*-fusion.

Cultures containing reporter plasmids were grown in either MOPS complete-glucose or MOPS-glucose, a defined minimal medium, overnight at 37°C and dilutions were performed to OD_420_ of 0.005 prior to the experiment in their respective mediums in triplicate. Once cultures had reached exponential growth samples of 500uL and 1mL were taken simultaneously to measure β-galactosidase (β-gal) activity and OD_420_ at three timepoints. β-gal assay samples were immediately added to a tube containing 500uL Z-buffer, 1.25uL beta-mercaptoethanol, 30uL 0.1% SDS and 40uL chloroform then vortexed before being placed on ice. Once collection was completed all β-gal sample tubes were incubated at 28°C prior to induction with 200uL 4mg/mL ONPG. Development was stopped with 500uL of 1M sodium bicarbonate and samples were centrifuged for 5 minutes at max speed to remove cellular debris. Samples were then transferred into a 96-well plate to measure OD_420_ in a Varioskan plate reader. Cell density and OD_420_ of developed β-gal samples were used to calculate Miller units of activity.

## Supporting information

S1 Fig Ribosome occupancy over stop codons. Metagene analysis of ribosome density for each three-base stop codon (UAA, UAG and UGA) and each four-base stop codon (each stop codon with a varying fourth position). Shown is the median ribosome footprint density in the region surrounding stop codons for K-12 RF2^K-12^ (4), K-12 RF2^K-12^ΔRF3 (2), K-12 RF2^B^ (2) and K-12 RF2^B^ΔRF3 (5) strains grown in MOPS-complete glucose medium at 37°C. Average normalized density was calculated across repeat experiments with the numbers in parentheses following each strain indicating the number of repeat experiments. In order to improve resolution for the rare UAG codon and across all possible four-base stop codons, we did not select genes with high read density; in total this dataset included 3390 genes.
(TIF)

S2 Fig Increased ribosome occupancy over stop codons of single genes. The normalized ribosome occupancy is shown over two randomly chosen genes, *torR* (A) and *dcrB* (B) for all strains; K-12 RF2^K-12^, K-12 RF2^B^, K-12 RF2^K-12^ΔRF3 and K-12 RF2^B^ΔRF3. The start codon of each gene is annotated with a green triangle and stop codon with a red triangle. Peak intensity between strains remains relatively consistent with the largest variability over the stop codon.
(TIF)

S3 Fig Ribosome occupancy over stop codons of genes with high translation efficiency. Metagene analysis of ribosome footprint density in the region surrounding stop codons. The top 10% of genes with the highest translation efficiency were aligned at their stop codon and the median normalized ribosome density at each position was calculated from ribosome profiling data of strains grown in MOPS complete-glucose media at 37°C. Translation efficiency was defined as the rate of protein production per mRNA molecule (RPKM of ribosome profiling reads normalized by RPKM of mRNA-seq reads) [43]. Average normalized density was calculated across repeat experiments for K-12 RF2^K-12^, K-12 RF2^K-12^ΔRF3, K-12 RF2^B^ and K-12 RF2^B^ΔRF3 containing 4, 2, 2 and 5 datasets respectively. In K-12 RF2^K-12^ΔRF3 a slight increase in ribosome occupancy is seen approximately 28nt upstream of the stop codon.
(TIF)

S4 Fig Post-ORF ribosome occupancy of ΔRF3 mutants for replicate experiments. (A) The cumulative distribution of RPOR values from 0 to 2.0 for K-12 RF2^K-12^ and K-12 RF2^K-12^ΔRF3 strains are shown for a replicate experiment to Fig 3B. After removal of all zero RPOR values, 163 genes were analyzed. The shift of K-12 RF2^K-12^ΔRF3 to higher RPOR values is statistically significant (p-value = 0.001; K-S test). (B) The distribution of RPOR values from 0 to 2.0 for K-12 RF2^K-12^ and two K-12 RF2^B^ΔRF3 replicates in the same experiment are shown using a cumulative distribution function. This analysis included 732 genes after all zero value RPORs were removed. The two K-12 RF2^B^ΔRF3 replicates are both statistically significant when compared to RF2^K-12^ (p-value < 0.005).
(TIF)

S5 Fig Post-ORF ribosome occupancy between RF2^B^ mutants. The cumulative distribution of relative post-ORF ribosome occupancy (RPOR) values between 0 and 2.0 of K-12 RF2^K-12^ and K-12 RF2^B^ for replicate experiments are shown. (A) Our largest dataset of 1139 genes analyzed shows a slight shift towards lower RPOR values in K-12 RF2^B^ versus K-12 RF2^K-12^, which is statistically significant (p-value = 5.3x10^-6^; K-S test). (B) A small dataset comprised of 163 genes analyzed, shows a slight statistically insignificant shift towards higher RPOR values for K-12 RF2^B^ versus K-12 RF2^K-12^.
(TIF)

S6 Fig Recoding at the *pheL* locus in all RF mutants. (A) Normalized ribosome footprints are shown across the locus of *pheL* (shaded green) and post-ORF region with hypothesized extensions shaded in red, which extend into *pheA*. Post-ORF stop codons are annotated for each reading frame with red bars; the known *pheL* +1 extension stop codon is marked with a red triangle within *pheA*. (A) A section of the *pheL* post-ORF region is enlarged and we see a reduction in ribosome density correlated with a 0-frame stop codon potentially signaling a readthrough event. (B) Western blots of α-FLAG and α-SurA (loading control, lc) for all strains. In addition to a non-specific binding product (n.s.) we observe full length PheL at 3.26kDa, and two possible extended PheL products, a 4.85kDa 0-frame product and a 9.0kDa consistent with a +1 frameshift. We estimate the +1 frameshift efficiency in K-12 RF2^K-12^ to be 52% and increases to 84% in K-12 RF2^K-12^ΔRF3. We do not observe any PheL products in either K-12 RF2^B^ strain; this is apparently consistent with the drastic shift in ribosome occupancy over *pheL* in K-12 RF2^B^ strains (A).
(TIF)

S7 Fig Western blots of C-terminal tag NudL constructs. Western blot of K-12 RF2^K-12^ΔRF3 strain containing a plasmid encoding N-terminal-FLAG NudL, with a C-terminal streptavidin tag on either the 0 frame or -1 frame extension. Membranes were blotted with both α-FLAG (A) and α-streptavidin (B). (A and B) FLAG tagged NudL products are seen in both 0 and -1 frame constructs, however only the 0 frame product blots for streptavidin, as indicated by the black arrow. The 0 frame construct was utilized for further studies.
(TIF)

S8 Fig Western blots of NudL and PanZ constructs in all strains. Western blot of α-FLAG and α-streptavidin for induced constructs of N-terminal-FLAG-NudL (A) or PanZ (B) in K-12 RF2^K-12^, K-12 RF2^B^, K-12 RF2^K-12^ΔRF3 and K-12 RF2^B^ΔRF3. SurA was used as a loading control (lc). (A) The streptavidin tagged extended NudL protein visible in K-12 RF2^K-12^ and K-12 RF2^K-12^ ΔRF3 is no longer visible in either K-12 RF2^B^ or K-12 RF2^B^ΔRF3. (B) The presence of the streptavidin tagged -1 frameshift extended PanZ product is seen in similar abundance in all strains, including K-12 RF2^B^ and K-12 RF2^B^ ΔRF3, at approximately 35% of total PanZ.
(TIF)

S9 Fig Translation of overlapping ORF pairs. The ratio of translation levels of overlapping ORFs [downstream (ORF2)/upstream (ORF1)] were compared between K-12 RF2^K-12^, K-12 RF2^K-12^ΔRF3, K-12 RF2^B^, and K-12 RF2^B^ΔRF3 strains. A total of 72 ORF pairs were analyzed. The translation level of each ORF was quantified by translation efficiency (TE), defined as the rate of protein production per mRNA molecule (RPKM of ribosome profiling reads normalized by RPKM of mRNA-seq read) [43]. The red diagonal lines represent equivalent expression of the two strains being compared. (A) Gene pairs with a TE ratio <0.5 have increased expression of downstream genes in the K-12 RF2^K-12^ΔRF3 strain, indicated by the dotted ellipse. (B and C) Compared to the K-12 RF2^K-12^ strain, theK-12 RF2^B^ ΔRF3 strain (B) and the K-12 RF2^B^ strain (C) show equivalent expression across all gene pairs.
(TIF)

S10 Fig Attenuation of *hisL* operon in ΔRF3. A reporter plasmid for *hisL* attenuation was constructed by fusing *lacZ* to the first gene downstream of the leader peptide, *hisG*. The promoter plasmid fused to the leader peptide used to normalize transcription from the operon promoter (see Fig 7C, reporter #1), was toxic under the control of the *hisL* promoter, and could not be used to normalize expression of the downstream gene reporter construct. We therefore report only the β-galactosidase activity of the downstream reporter in Miller units for K-12 RF2^K-12^ and K-12 RF2^K-12^ΔRF3 backgrounds in MOPS-complete glucose and MOPS-minimal glucose media.
(TIF)

S1 Table Annotated likely recoding events. Of the top 100 post-ORF ribosome occupancy (RPOR) values in K-12 RF2^K-12^ and K-12 RF2^K-12^ΔRF3 (121 total), 43 were classified as likely recoding events because they experienced reduced ribosome density following a stop codon in any one of the three possible reading frames, a proxy for active post-ORF translation (Fig 5). The RPOR value for strains; K-12 RF2^K-12^, K-12 RF2^K-12^ΔRF3, K-12 RF2^B^ and K-12 RF2^B^ΔRF3 are shown for each of the genes. The genes with asterisk (*) indicate those used in follow-up studies with N-terminal-FLAG, C-terminal-streptavidin constructs to visualize extended products.
(DOCX)

S2 Table Annotated possible recoding events. Of the top 100 post-ORF ribosome occupancy (RPOR) values in K-12 RF2^K-12^ and K-12 RF2^K-12^ΔRF3 strains (121 total), 41 were classified as possible recoding events because in addition to the reduction in ribosome occupancy after a stop codon described above (Fig 5), possible confounding effects indicated in the column labeled “class” were present. They are annotated as; a: unannotated downstream ORF; b: REP-element in the post-ORF region; c: Shine-Dalgarno (SD) or downstream gene is located within the post-ORF region; d: ribosomes from another unknown source; e: low reads in the post-ORF region.
(DOCX)

S3 Table Annotated non-recoding events. Of the top 100 post-ORF ribosome occupancy (RPOR) values in K-12 RF2^K-12^ and K-12 RF2^K-12^ΔRF3 strains (121 total), 37 were classified as non-recoding events. The post-ORF region of these genes did not exhibit a reduction in ribosome density after stop codons in any frame suggestive that ribosome occupancy was not due to active translation (Fig 5). Sequence elements of these post-ORF region and possible sources of these post-ORF ribosomes were annotated in column labeled “class” as; a: small RNA; b: REP-element c: Shine-Dalgarno (SD) or downstream gene; d: ribosomes from an unknown source; e: ribosome binding region.
(DOCX)

S4 Table Misannotations resulting in high RPOR values. Of the top 100 post-ORF ribosome occupancy (RPOR) values in K-12 RF2^K-12^ and K-12 RF2^K-12^ΔRF3 strains (121 total), four instances result from misannotations. The post-ORF region of the genes listed was located over an unidentified or recently identified ORF.
(DOCX)

S5 Table Stop codon distribution of annotated recoding events. The distribution of stop codons of the 84 genes classified as likely or possible recoding events in S1 and S2 Tables is shown in the observed column. The expected values were calculated using the frequencies in which each codon, UAA, UAG and UGA occur across the *E*.*coli* K-12 genome. The Chi-squared value was calculated to be 26.277 with 2 degrees of freedom, which is statistically significant by two-tailed t-test with a p-value of less than 0.0001.
(DOCX)
