# Supplementary figures and images for: Global analysis of translation termination in E. coli
Source: PLoS Genet. 2017 Mar 16;13(3):e1006676. doi: 10.1371/journal.pgen.1006676 (PMC5373646; doi:10.1371/journal.pgen.1006676)

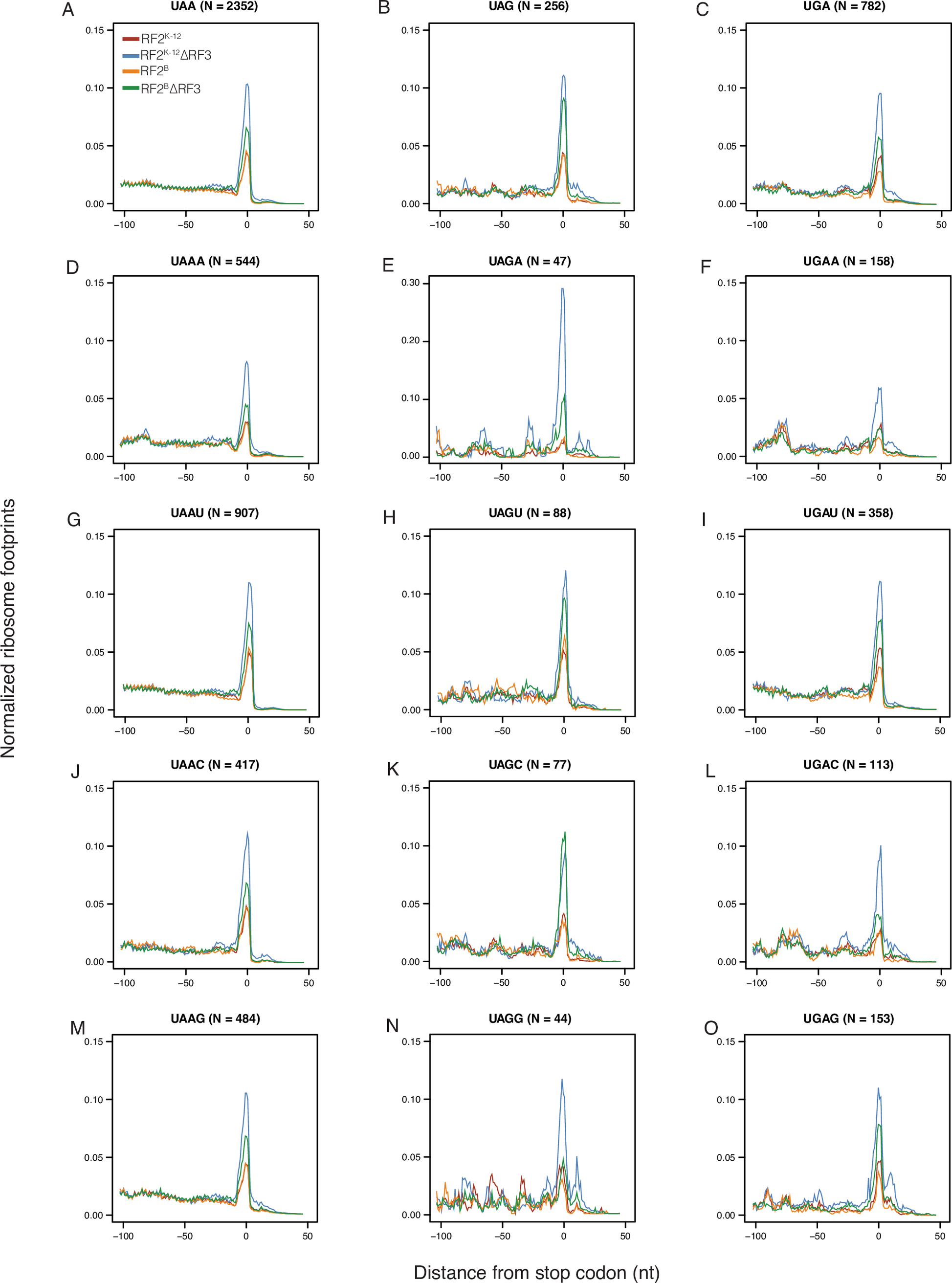

Supplement: S1 Fig — Metagene analysis of ribosome density for each three-base stop codon (UAA, UAG and UGA) and each four-base stop codon (each stop codon with a varying fourth position). Shown is the median ribosome footprint density in the region surrounding stop codons for K-12 RF2K-12 (4), K-12 RF2K-12ΔRF3 (2), K-12 RF2B (2) and K-12 RF2BΔRF3 (5) strains grown in MOPS-complete glucose medium at 37°C. Average normalized density was calculated across repeat experiments with the numbers in parentheses following each strain indicating the number of repeat experiments. In order to improve resolution for the rare UAG codon and across all possible four-base stop codons, we did not select genes with high read density; in total this dataset included 3390 genes. (TIF) [file pgen.1006676.s001.tif]

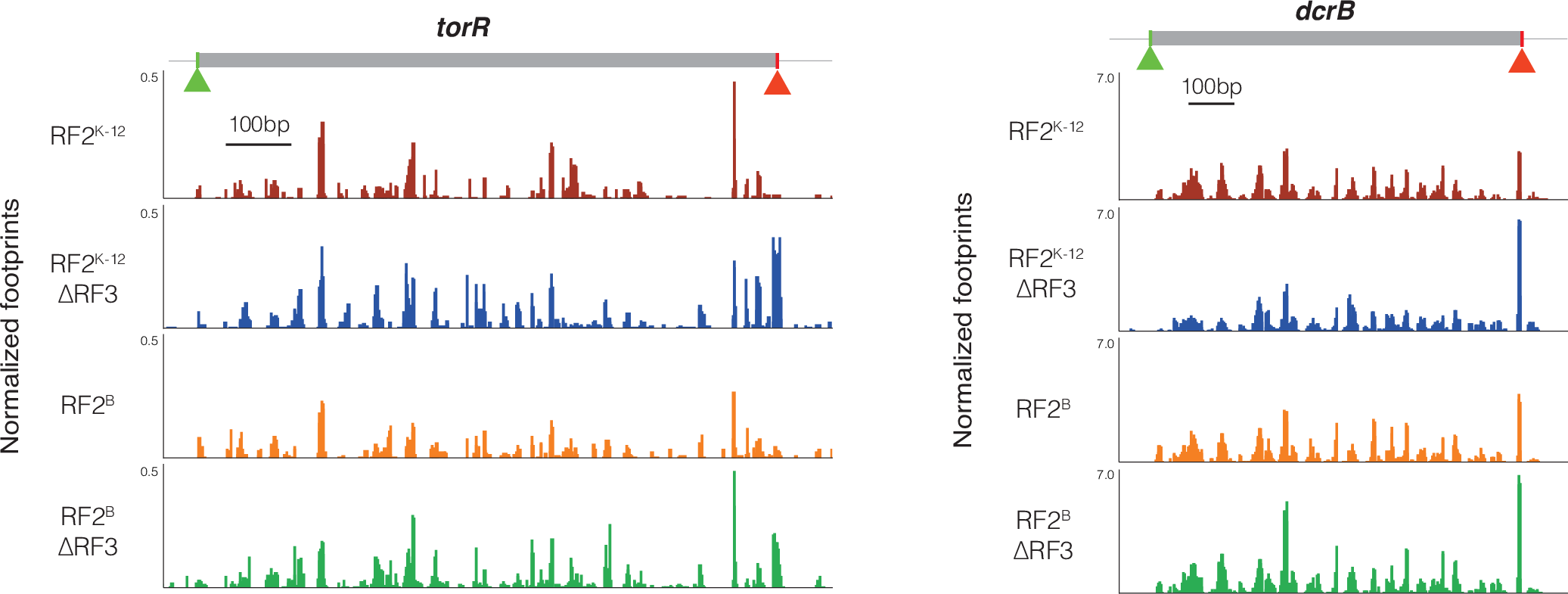

Supplement: S2 Fig — The normalized ribosome occupancy is shown over two randomly chosen genes, torR (A) and dcrB (B) for all strains; K-12 RF2K-12, K-12 RF2B, K-12 RF2K-12ΔRF3 and K-12 RF2BΔRF3. The start codon of each gene is annotated with a green triangle and stop codon with a red triangle. Peak intensity between strains remains relatively consistent with the largest variability over the stop codon. (TIF) [file pgen.1006676.s002.tif]

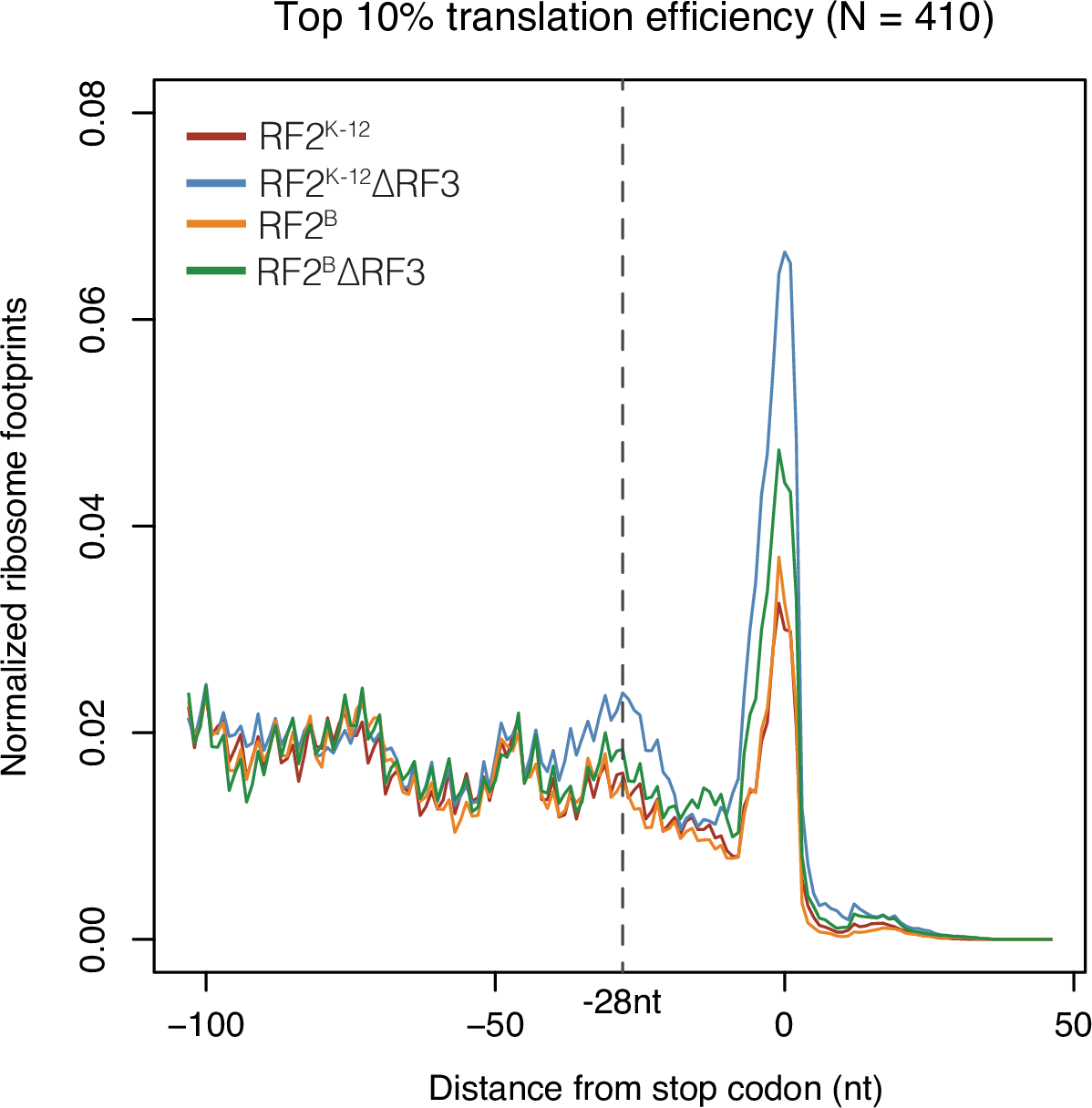

Supplement: S3 Fig — Metagene analysis of ribosome footprint density in the region surrounding stop codons. The top 10% of genes with the highest translation efficiency were aligned at their stop codon and the median normalized ribosome density at each position was calculated from ribosome profiling data of strains grown in MOPS complete-glucose media at 37°C. Translation efficiency was defined as the rate of protein production per mRNA molecule (RPKM of ribosome profiling reads normalized by RPKM of mRNA-seq reads) [43]. Average normalized density was calculated across repeat experiments for K-12 RF2K-12, K-12 RF2K-12ΔRF3, K-12 RF2B and K-12 RF2BΔRF3 containing 4, 2, 2 and 5 datasets respectively. In K-12 RF2K-12ΔRF3 a slight increase in ribosome occupancy is seen approximately 28nt upstream of the stop codon. (TIF) [file pgen.1006676.s003.tif]

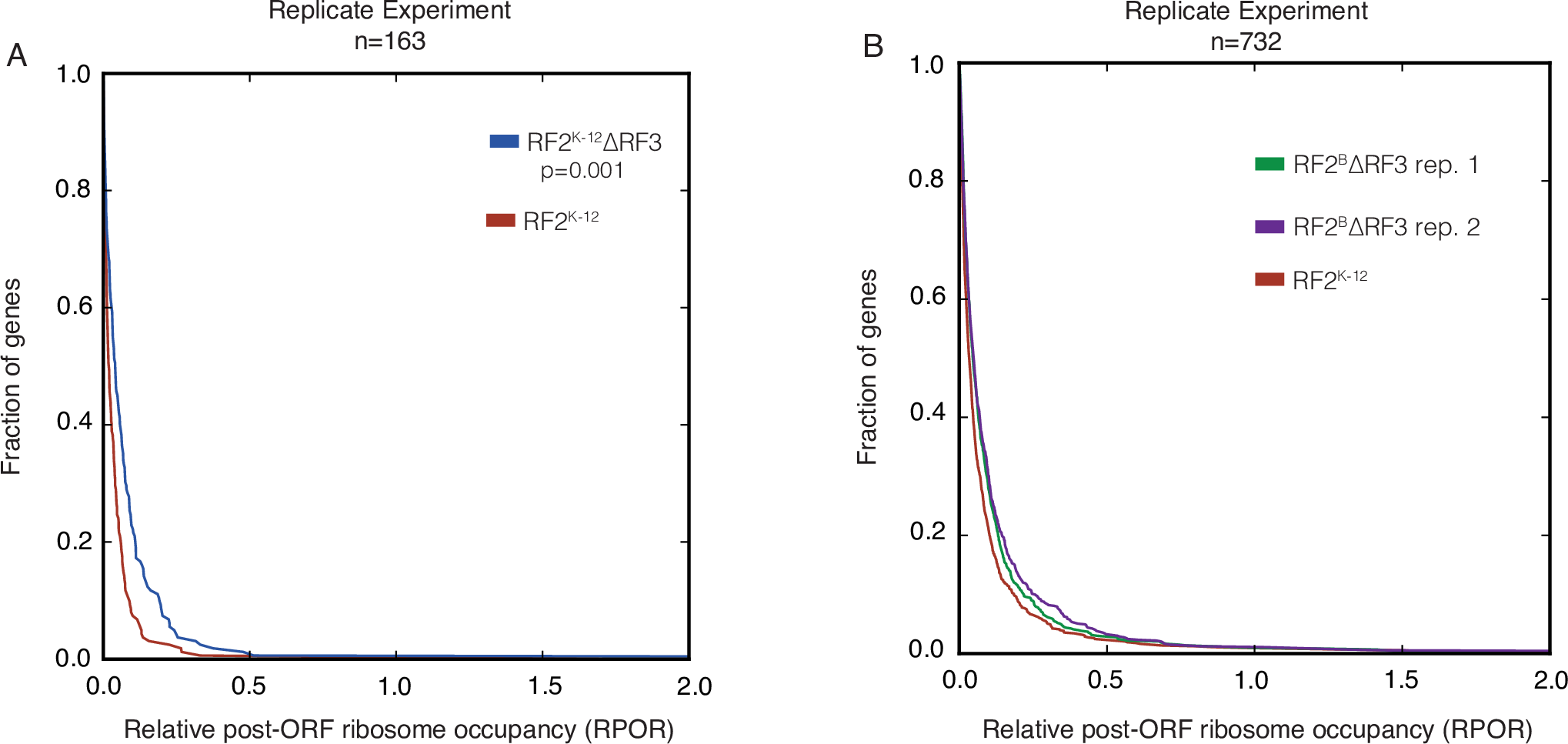

Supplement: S4 Fig — (A) The cumulative distribution of RPOR values from 0 to 2.0 for K-12 RF2K-12 and K-12 RF2K-12ΔRF3 strains are shown for a replicate experiment to Fig 3B. After removal of all zero RPOR values, 163 genes were analyzed. The shift of K-12 RF2K-12ΔRF3 to higher RPOR values is statistically significant (p-value = 0.001; K-S test). (B) The distribution of RPOR values from 0 to 2.0 for K-12 RF2K-12 and two K-12 RF2BΔRF3 replicates in the same experiment are shown using a cumulative distribution function. This analysis included 732 genes after all zero value RPORs were removed. The two K-12 RF2BΔRF3 replicates are both statistically significant when compared to RF2K-12 (p-value < 0.005). (TIF) [file pgen.1006676.s004.tif]

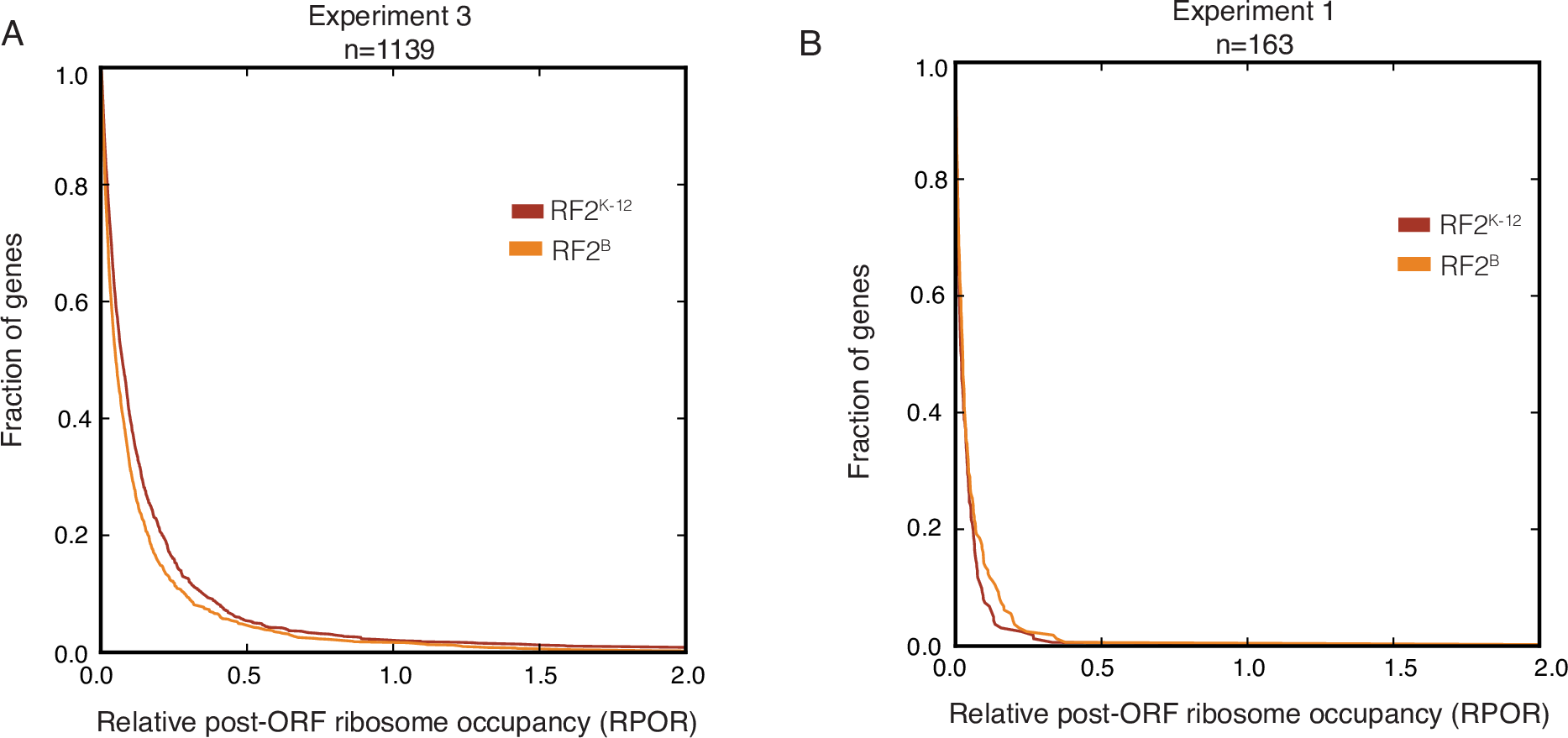

Supplement: S5 Fig — The cumulative distribution of relative post-ORF ribosome occupancy (RPOR) values between 0 and 2.0 of K-12 RF2K-12 and K-12 RF2B for replicate experiments are shown. (A) Our largest dataset of 1139 genes analyzed shows a slight shift towards lower RPOR values in K-12 RF2B versus K-12 RF2K-12, which is statistically significant (p-value = 5.3x10^-6^; K-S test). (B) A small dataset comprised of 163 genes analyzed, shows a slight statistically insignificant shift towards higher RPOR values for K-12 RF2B versus K-12 RF2K-12. (TIF) [file pgen.1006676.s005.tif]

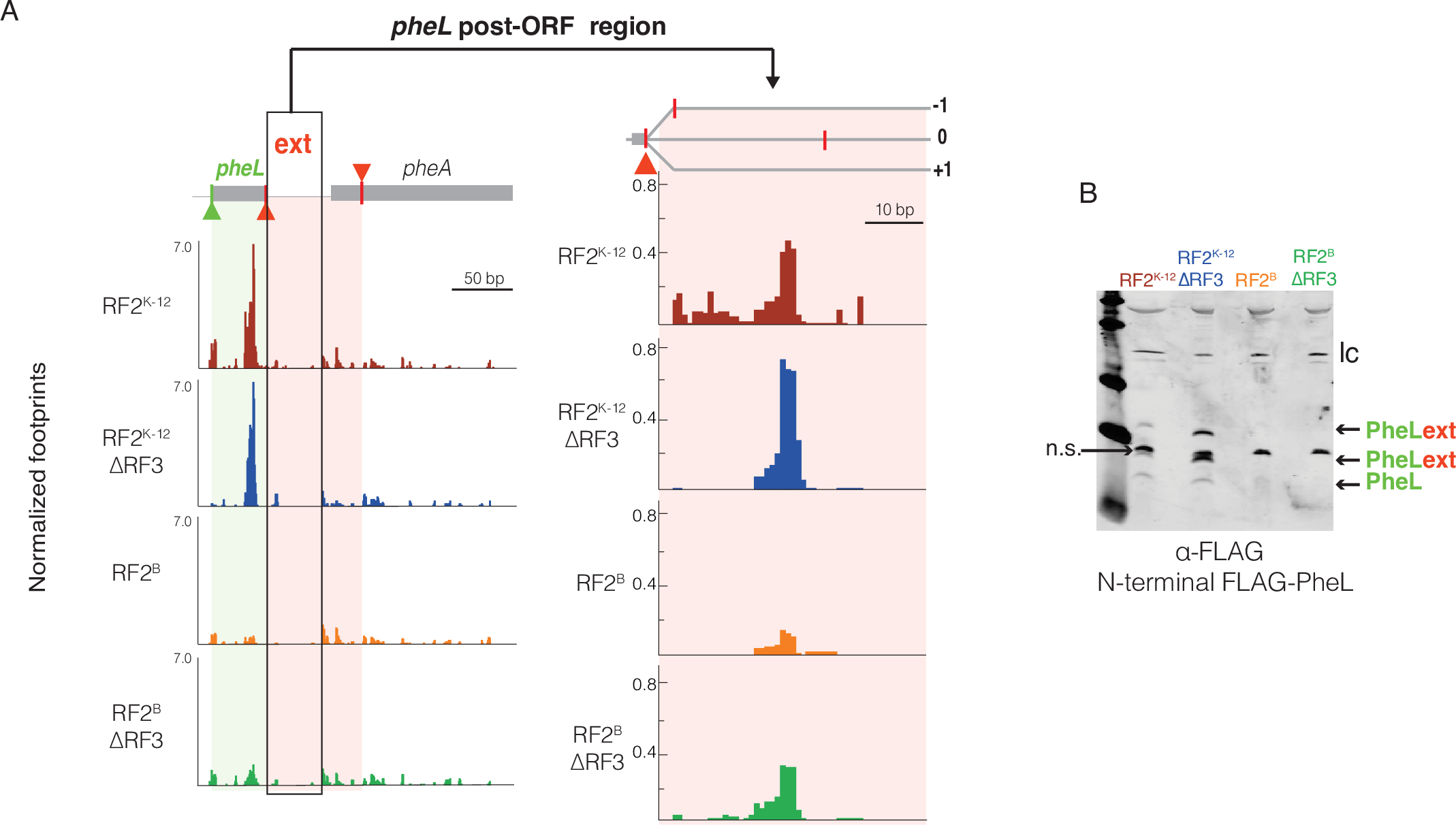

Supplement: S6 Fig — (A) Normalized ribosome footprints are shown across the locus of pheL (shaded green) and post-ORF region with hypothesized extensions shaded in red, which extend into pheA. Post-ORF stop codons are annotated for each reading frame with red bars; the known pheL +1 extension stop codon is marked with a red triangle within pheA. (A) A section of the pheL post-ORF region is enlarged and we see a reduction in ribosome density correlated with a 0-frame stop codon potentially signaling a readthrough event. (B) Western blots of α-FLAG and α-SurA (loading control, lc) for all strains. In addition to a non-specific binding product (n.s.) we observe full length PheL at 3.26kDa, and two possible extended PheL products, a 4.85kDa 0-frame product and a 9.0kDa consistent with a +1 frameshift. We estimate the +1 frameshift efficiency in K-12 RF2K-12 to be 52% and increases to 84% in K-12 RF2K-12ΔRF3. We do not observe any PheL products in either K-12 RF2B strain; this is apparently consistent with the drastic shift in ribosome occupancy over pheL in K-12 RF2B strains (A). (TIF) [file pgen.1006676.s006.tif]

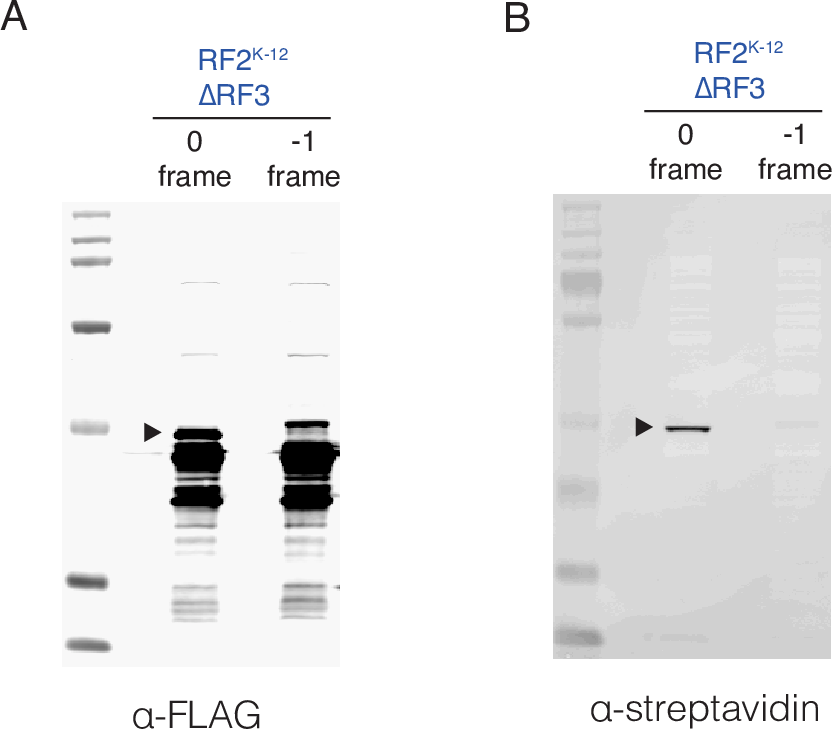

Supplement: S7 Fig — Western blot of K-12 RF2K-12ΔRF3 strain containing a plasmid encoding N-terminal-FLAG NudL, with a C-terminal streptavidin tag on either the 0 frame or -1 frame extension. Membranes were blotted with both α-FLAG (A) and α-streptavidin (B). (A and B) FLAG tagged NudL products are seen in both 0 and -1 frame constructs, however only the 0 frame product blots for streptavidin, as indicated by the black arrow. The 0 frame construct was utilized for further studies. (TIF) [file pgen.1006676.s007.tif]

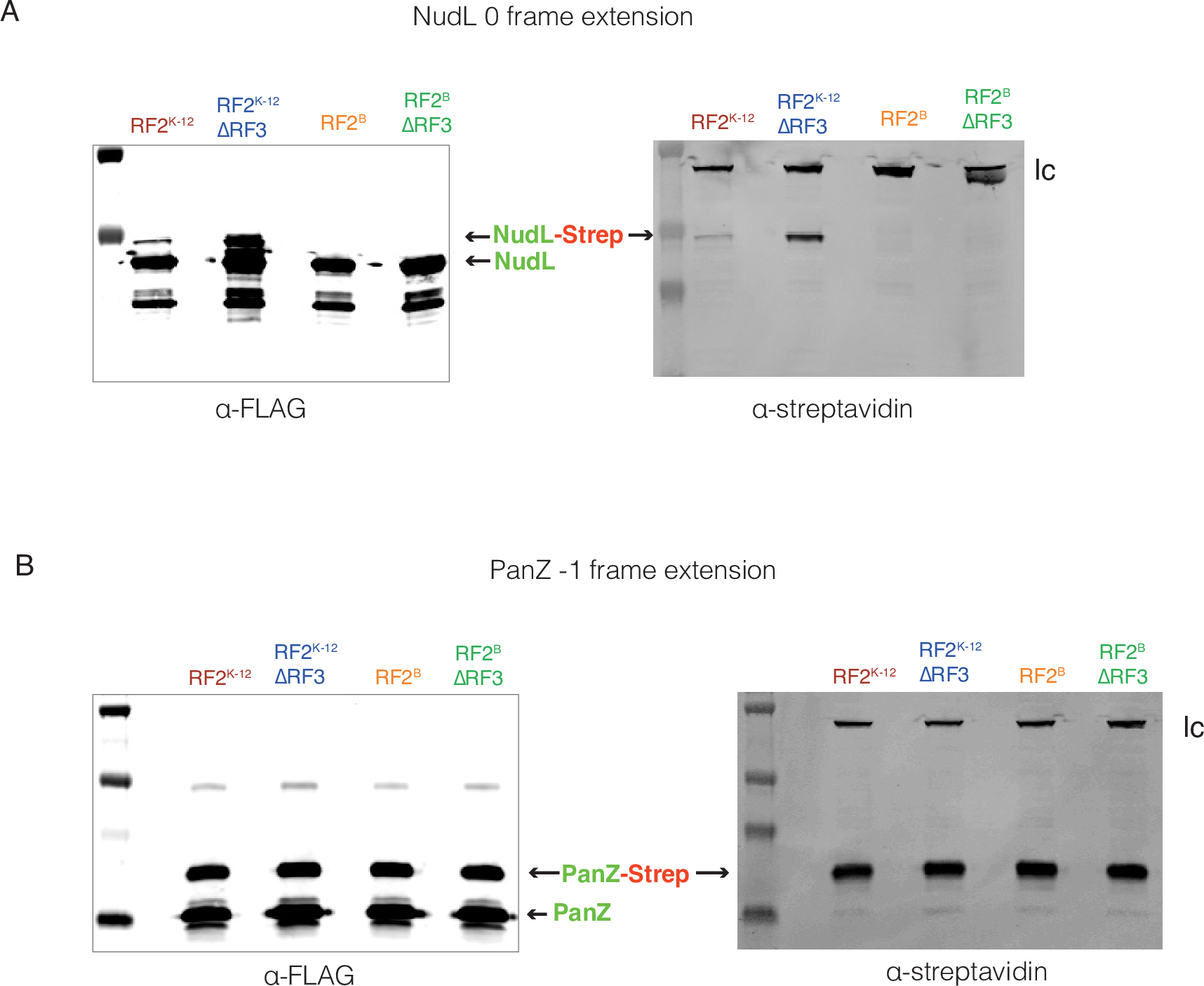

Supplement: S8 Fig — Western blot of α-FLAG and α-streptavidin for induced constructs of N-terminal-FLAG-NudL (A) or PanZ (B) in K-12 RF2K-12, K-12 RF2B, K-12 RF2K-12ΔRF3 and K-12 RF2BΔRF3. SurA was used as a loading control (lc). (A) The streptavidin tagged extended NudL protein visible in K-12 RF2K-12 and K-12 RF2K-12 ΔRF3 is no longer visible in either K-12 RF2B or K-12 RF2BΔRF3. (B) The presence of the streptavidin tagged -1 frameshift extended PanZ product is seen in similar abundance in all strains, including K-12 RF2B and K-12 RF2B ΔRF3, at approximately 35% of total PanZ. (TIF) [file pgen.1006676.s008.tif]

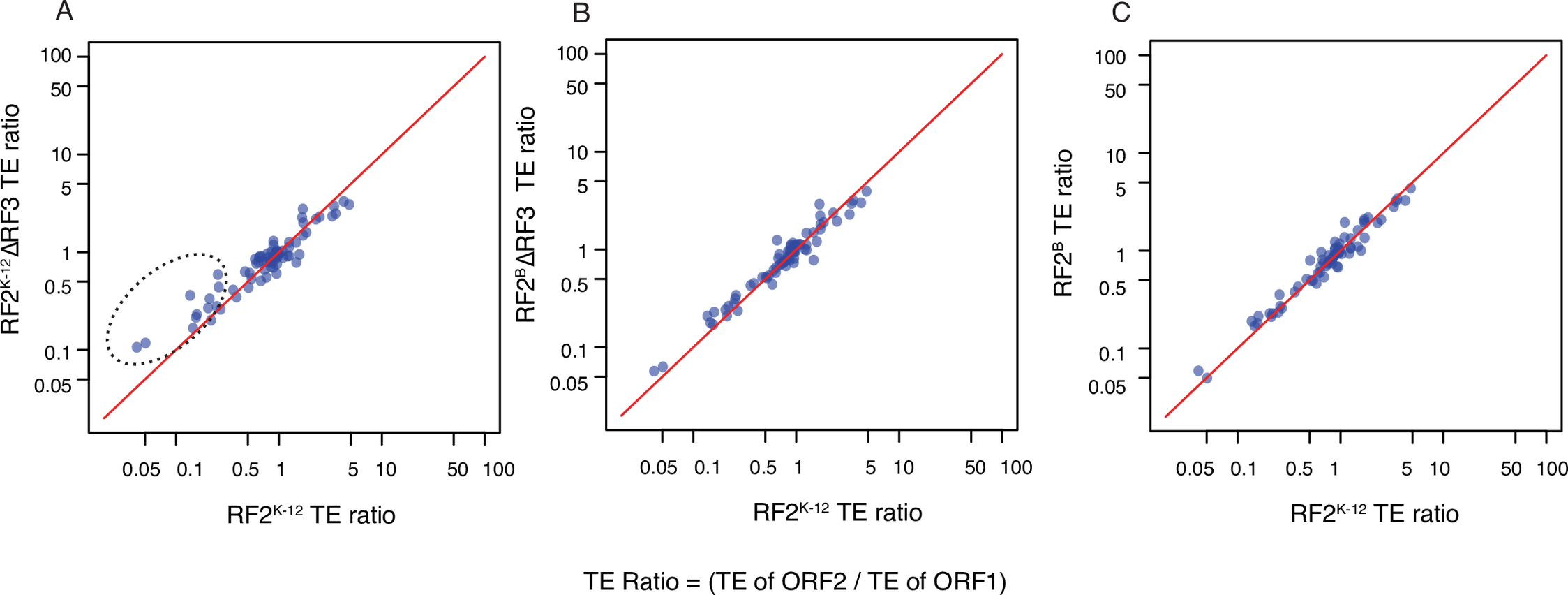

Supplement: S9 Fig — The ratio of translation levels of overlapping ORFs [downstream (ORF2)/upstream (ORF1)] were compared between K-12 RF2K-12, K-12 RF2K-12ΔRF3, K-12 RF2B, and K-12 RF2BΔRF3 strains. A total of 72 ORF pairs were analyzed. The translation level of each ORF was quantified by translation efficiency (TE), defined as the rate of protein production per mRNA molecule (RPKM of ribosome profiling reads normalized by RPKM of mRNA-seq read) [43]. The red diagonal lines represent equivalent expression of the two strains being compared. (A) Gene pairs with a TE ratio <0.5 have increased expression of downstream genes in the K-12 RF2K-12ΔRF3 strain, indicated by the dotted ellipse. (B and C) Compared to the K-12 RF2K-12 strain, theK-12 RF2B ΔRF3 strain (B) and the K-12 RF2B strain (C) show equivalent expression across all gene pairs. (TIF) [file pgen.1006676.s009.tif]

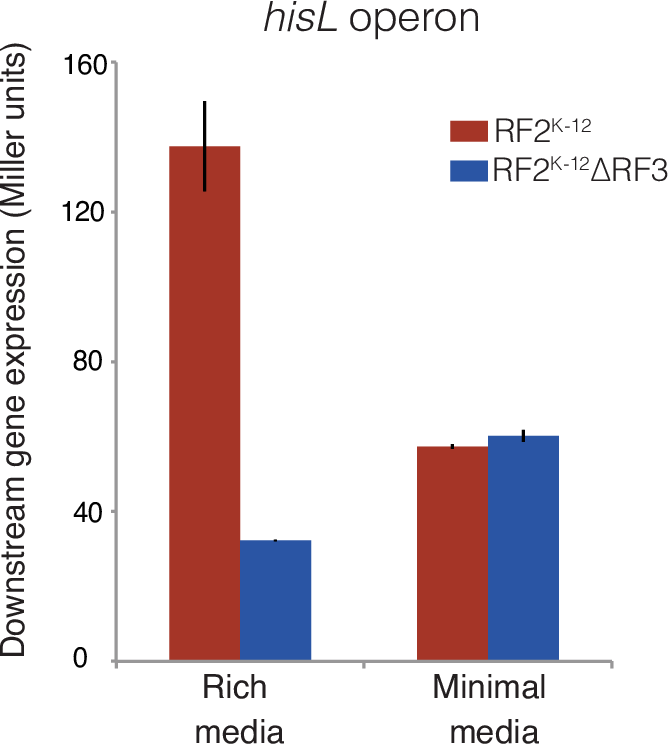

Supplement: S10 Fig — A reporter plasmid for hisL attenuation was constructed by fusing lacZ to the first gene downstream of the leader peptide, hisG. The promoter plasmid fused to the leader peptide used to normalize transcription from the operon promoter (see Fig 7C, reporter #1), was toxic under the control of the hisL promoter, and could not be used to normalize expression of the downstream gene reporter construct. We therefore report only the β-galactosidase activity of the downstream reporter in Miller units for K-12 RF2K-12 and K-12 RF2K-12ΔRF3 backgrounds in MOPS-complete glucose and MOPS-minimal glucose media. (TIF) [file pgen.1006676.s010.tif]
