# Supplementary material for: Global analysis of translation termination in E. coli
Source: PLoS Genet. 2017 Mar 16;13(3):e1006676. doi: 10.1371/journal.pgen.1006676 (PMC5373646; doi:10.1371/journal.pgen.1006676)
Supplement: S1 Table — Of the top 100 post-ORF ribosome occupancy (RPOR) values in K-12 RF2K-12 and K-12 RF2K-12ΔRF3 (121 total), 43 were classified as likely recoding events because they experienced reduced ribosome density following a stop codon in any one of the three possible reading frames, a proxy for active post-ORF translation (Fig 5). The RPOR value for strains; K-12 RF2K-12, K-12 RF2K-12ΔRF3, K-12 RF2B and K-12 RF2BΔRF3 are shown for each of the genes. The genes with asterisk (*) indicate those used in follow-up studies with N-terminal-FLAG, C-terminal-streptavidin constructs to visualize extended products. (DOCX) [file pgen.1006676.s011.docx]

| gene | RF2^K-12^  RPOR | RF2^K-12^  ∆RF3  RPOR | RF2^B^  RPOR | RF2^B^∆RF3  RPOR |
| --- | --- | --- | --- | --- |
| ***ydbL*** | 5.75 | 4.65 | 2.33 | 3.42 |
| ***ybbB*** | 2.31 | 4.14 | 1.21 | 1.86 |
| ***ydcA*** | 1.91 | 3.11 | 1.43 | 1.78 |
| ***nadR*** | 1.73 | 2.63 | 1.51 | 1.85 |
| ***barA*** | 1.58 | 6.80 | 0.63 | 0.23 |
| ***yegH*** | 1.09 | 2.06 | 1.24 | 1.18 |
| ***yiaU*** | 1.00 | 2.69 | 0.80 | 0.72 |
| ***nudL**** | 0.89 | 0.93 | 1.20 | 0.81 |
| ***yhiN*** | 0.83 | 3.66 | 1.75 | 1.54 |
| ***yneE*** | 0.80 | 0.89 | 0.44 | 1.29 |
| ***setA*** | 0.75 | 0.84 | 0.09 | 0.46 |
| ***ycbB*** | 0.71 | 2.70 | 0.59 | 0.86 |
| ***betA*** | 0.64 | 1.64 | 0.60 | 1.14 |
| ***ybiU*** | 0.64 | 1.61 | 0.66 | 0.76 |
| ***yidQ*** | 0.63 | 0.91 | 0.09 | 0.20 |
| ***hcaT*** | 0.57 | 0.74 | 0.44 | 0.72 |
| ***ybhK*** | 0.56 | 0.31 | 0.64 | 0.36 |
| ***pheL**** | 0.51 | 1.10 | 0.41 | 0.72 |
| ***ydiH*** | 0.49 | 0.80 | 0.64 | 1.22 |
| ***yjaB*** | 0.48 | 0.67 | 0.05 | 0.02 |
| ***yneG*** | 0.47 | 0.52 | 0.29 | 0.27 |
| ***ydgC*** | 0.46 | 0.16 | 0.37 | 0.60 |
| ***udk*** | 0.46 | 1.21 | 0.48 | 0.67 |
| ***flk*** | 0.45 | 0.52 | 0.38 | 0.50 |
| ***argO*** | 0.44 | 0.69 | 0.25 | 0.27 |
| ***fhuF*** | 0.43 | 0.64 | 0.30 | 0.12 |
| ***panZ**** | 0.42 | 0.96 | 0.54 | 0.47 |
| ***dbpA*** | 0.42 | 0.64 | 0.19 | 0.48 |
| ***pdxH*** | 0.41 | 0.35 | 0.39 | 0.64 |
| ***yadS*** | 0.41 | 1.24 | 0.14 | 0.33 |
| ***pspG*** | 0.39 | 1.93 | 0.42 | 0.60 |
| ***dusC*** | 0.38 | 0.89 | 0.59 | 0.82 |
| ***yiaJ*** | 0.34 | 0.78 | 0.11 | 0.56 |
| ***aat*** | 0.32 | 1.98 | 0.86 | 1.25 |
| ***zwf*** | 0.27 | 0.65 | 0.15 | 0.22 |
| ***yafJ*** | 0.26 | 0.97 | 0.33 | 0.19 |
| ***obgE*** | 0.26 | 0.62 | 0.21 | 0.35 |
| ***yidE*** | 0.23 | 0.66 | 0.23 | 0.08 |
| ***mpaA*** | 0.21 | 0.70 | 0.00 | 0.23 |
| ***ygiH*** | 0.12 | 0.73 | 0.13 | 0.14 |
| ***dapB*** | 0.12 | 0.75 | 0.16 | 0.25 |
| ***dsdC*** | 0.08 | 0.66 | 0.17 | 0.10 |
| ***ydiA*** | 0.02 | 0.66 | 0.16 | 0.46 |
