# Supplementary material for: Global analysis of translation termination in E. coli
Source: PLoS Genet. 2017 Mar 16;13(3):e1006676. doi: 10.1371/journal.pgen.1006676 (PMC5373646; doi:10.1371/journal.pgen.1006676)
Supplement: S2 Table — Of the top 100 post-ORF ribosome occupancy (RPOR) values in K-12 RF2K-12 and K-12 RF2K-12ΔRF3 strains (121 total), 41 were classified as possible recoding events because in addition to the reduction in ribosome occupancy after a stop codon described above (Fig 5), possible confounding effects indicated in the column labeled “class” were present. They are annotated as; a: unannotated downstream ORF; b: REP-element in the post-ORF region; c: Shine-Dalgarno (SD) or downstream gene is located within the post-ORF region; d: ribosomes from another unknown source; e: low reads in the post-ORF region. (DOCX) [file pgen.1006676.s012.docx]

| gene | RF2^K-12^  RPOR | RF2^K-12^  ∆RF3  RPOR | RF2^B^  RPOR | RF2^B^∆RF3  RPOR | class |
| --- | --- | --- | --- | --- | --- |
| ***ykgG*** | 3.21 | 13.53 | 6.29 | 3.12 | a |
| ***xapR*** | 2.06 | 5.24 | 1.07 | 1.41 | b & e |
| ***yjdP*** | 1.51 | 1.22 | 1.09 | 1.10 | e |
| ***cusS*** | 1.47 | 2.02 | 0.67 | 5.62 | e |
| ***yabP*** | 1.29 | 2.53 | 0.47 | 0.55 | c |
| ***pspD*** | 1.21 | 4.41 | 0.56 | 0.88 | c |
| ***chbG*** | 1.03 | 1.90 | 0.67 | 1.08 | b & e |
| ***polB*** | 0.97 | 2.05 | 0.56 | 1.09 | e |
| ***cvrA*** | 0.89 | 0.51 | 0.51 | 0.37 | e |
| ***ftsK*** | 0.89 | 1.00 | 0.52 | 1.13 | b |
| ***yaiA*** | 0.87 | 1.14 | 0.50 | 0.77 | c |
| ***ypfI*** | 0.83 | 1.22 | 0.82 | 2.15 | e |
| ***recC*** | 0.81 | 0.71 | 0.66 | 0.94 | e |
| ***intZ*** | 0.78 | 0.61 | 0.32 | 0.26 | c |
| ***yidZ*** | 0.69 | 3.53 | 1.05 | 3.01 | e |
| ***yiaG*** | 0.63 | 0.19 | 0.46 | 0.84 | e |
| ***hipA*** | 0.56 | 0.33 | 0.15 | 0.34 | d |
| ***yabP*** | 0.56 | 0.95 | 0.47 | 0.55 | c |
| ***yeeA*** | 0.55 | 0.47 | 0.21 | 0.54 | c |
| ***rmuC*** | 0.55 | 0.25 | 0.52 | 0.16 | e |
| ***fre*** | 0.52 | 0.53 | 0.07 | 0.10 | b |
| ***yjdJ*** | 0.52 | 0.19 | 0.17 | 0.13 | e |
| ***mltA*** | 0.49 | 0.64 | 0.65 | 1.00 | c |
| ***hpf*** | 0.49 | 0.26 | 0.29 | 0.45 | c |
| ***pitA*** | 0.46 | 0.31 | 0.22 | 0.31 | c |
| ***yhbQ*** | 0.45 | 1.05 | 0.78 | 0.78 | e |
| ***rcsC*** | 0.44 | 0.19 | 0.49 | 0.38 | e |
| ***rrmJ*** | 0.44 | 0.07 | 0.15 | 0.22 | e |
| ***yfiE*** | 0.42 | 0.63 | 0.08 | 0.23 | e |
| ***cobC*** | 0.366 | 1.138 | 1.42 | 0.91 | d |
| ***sbcC*** | 0.317 | 2.273 | 0.27 | 0.58 | d |
| ***mglC*** | 0.301 | 0.709 | 0.25 | 0.34 | e |
| ***xylR*** | 0.275 | 0.841 | 1.55 | 0.45 | b & e |
| ***pspF*** | 0.257 | 0.758 | 0.12 | 0.47 | c |
| ***gadX*** | 0.241 | 0.907 | 0.07 | 0.83 | e |
| ***eptB*** | 0.200 | 0.999 | 0.24 | 0.41 | d |
| ***cueR*** | 0.179 | 1.166 | 0.13 | 0.39 | d |
| ***uspB*** | 0.144 | 0.845 | 0.00 | 0.46 | e |
| ***livF*** | 0.110 | 0.971 | 0.05 | 0.34 | c |
| ***appY*** | 0.098 | 1.660 | 0.17 | 1.24 | e |
| ***yhdP*** | 0.080 | 0.962 | 0.37 | 0.64 | c |
| ***yddE*** | 0.075 | 1.157 | 0.00 | 0.17 | b & e |
| ***yhgE*** | 0.000 | 0.781 | 0.74 | 0.94 | e |

Classes:

a: unannotated downstream element

b: REP-element

c: Shine-Dalgarno (SD)

d: ribosomes from another unknown source

e: low reads within the post-ORF region
