# Supplementary material for: Global analysis of translation termination in E. coli
Source: PLoS Genet. 2017 Mar 16;13(3):e1006676. doi: 10.1371/journal.pgen.1006676 (PMC5373646; doi:10.1371/journal.pgen.1006676)
Supplement: S3 Table — Of the top 100 post-ORF ribosome occupancy (RPOR) values in K-12 RF2K-12 and K-12 RF2K-12ΔRF3 strains (121 total), 37 were classified as non-recoding events. The post-ORF region of these genes did not exhibit a reduction in ribosome density after stop codons in any frame suggestive that ribosome occupancy was not due to active translation (Fig 5). Sequence elements of these post-ORF region and possible sources of these post-ORF ribosomes were annotated in column labeled “class” as; a: small RNA; b: REP-element c: Shine-Dalgarno (SD) or downstream gene; d: ribosomes from an unknown source; e: ribosome binding region. (DOCX) [file pgen.1006676.s013.docx]

| gene | RF2^K-12^  RPOR | RF2^K-12^  ∆RF3  RPOR | RF2^B^  RPOR | RF2^B^∆RF3  RPOR | class |
| --- | --- | --- | --- | --- | --- |
| ***sgrR*** | 2.16 | 1.49 | 1.12 | 1.07 | a |
| ***yifN*** | 2.05 | 0.74 | 0.95 | 0.71 | a |
| ***yiiQ*** | 2.04 | 1.09 | 1.23 | 0.98 | c |
| ***yjdC*** | 1.34 | 0.42 | 0.45 | 0.47 | d |
| ***clcA*** | 1.27 | 1.49 | 0.84 | 0.98 | c |
| ***ybjN*** | 1.10 | 1.18 | 0.32 | 0.36 | d |
| ***iclR*** | 0.89 | 0.61 | 0.22 | 0.25 | c |
| ***htrL*** | 0.79 | 0.47 | 0.55 | 0.82 | d |
| ***ybjL*** | 0.70 | 0.44 | 0.17 | 0.25 | d |
| ***rpmJ*** | 0.69 | 0.13 | 0.41 | 0.62 | e |
| ***btuD*** | 0.64 | 0.38 | 0.30 | 0.78 | c |
| ***mtfA*** | 0.62 | 1.86 | 1.29 | 2.08 | d |
| ***ycjG*** | 0.62 | 0.37 | 0.35 | 0.18 | d |
| ***yqaA*** | 0.57 | 0.90 | 0.87 | 0.40 | c |
| ***ybbN*** | 0.55 | 0.67 | 0.62 | 0.66 | b |
| ***rnd*** | 0.55 | 0.15 | 0.22 | 0.24 | d |
| ***cmk*** | 0.53 | 0.60 | 0.46 | 0.27 | e |
| ***yjeT*** | 0.52 | 0.62 | 0.47 | 0.56 | d |
| ***pyrG*** | 0.48 | 0.20 | 0.23 | 0.39 | d |
| ***cpxP*** | 0.48 | 1.29 | 0.41 | 0.44 | a |
| ***infC*** | 0.45 | 0.22 | 0.32 | 0.38 | d |
| ***ycgX*** | 0.44 | 1.73 | 1.11 | 1.27 | d |
| ***ycgJ*** | 0.43 | 0.62 | 0.41 | 0.49 | d |
| ***yfbR*** | 0.43 | 0.10 | 0.22 | 0.33 | b |
| ***tolA*** | 0.42 | 0.27 | 0.21 | 0.32 | d |
| ***ynfA*** | 0.42 | 0.21 | 0.58 | 0.34 | d |
| ***mobB*** | 0.41 | 0.13 | 0.05 | 0.52 | b |
| ***iap*** | 0.41 | 0.06 | 0.08 | 0.59 | d |
| ***ycaK*** | 0.33 | 0.65 | 0.25 | 0.86 | d |
| ***ygjR*** | 0.33 | 0.76 | 0.04 | 0.22 | d |
| ***uup*** | 0.32 | 0.65 | 0.20 | 0.31 | c |
| ***csrD*** | 0.24 | 0.64 | 0.34 | 0.53 | d |
| ***cho*** | 0.23 | 0.74 | 0.44 | 1.00 | c |
| ***araC*** | 0.22 | 0.74 | 0.25 | 0.18 | d |
| ***yfcO*** | 0.20 | 0.73 | 0.12 | 0.54 | c |
| ***yhjJ*** | 0.11 | 0.62 | 0.24 | 0.34 | b |
| ***bssS*** | 0.09 | 1.96 | 0.17 | 0.54 | c |

Classes:

a: small RNA

b: REP-element

c: Shine-Dalgarno (SD)

d: ribosomes from another unknown source

e: ribosome binding region
