# Supplementary material for: Global analysis of translation termination in E. coli
Source: PLoS Genet. 2017 Mar 16;13(3):e1006676. doi: 10.1371/journal.pgen.1006676 (PMC5373646; doi:10.1371/journal.pgen.1006676)
Supplement: S4 Table — Of the top 100 post-ORF ribosome occupancy (RPOR) values in K-12 RF2K-12 and K-12 RF2K-12ΔRF3 strains (121 total), four instances result from misannotations. The post-ORF region of the genes listed was located over an unidentified or recently identified ORF. (DOCX) [file pgen.1006676.s014.docx]

| gene | RF2^K-12^  RPOR | RF2^K-12^  ∆RF3  RPOR | RF2^B^  RPOR | RF2^B^∆RF3  RPOR |
| --- | --- | --- | --- | --- |
| ***ydcM*** | 4.07 | 6.70 | 4.80 | 7.68 |
| ***yeaP*** | 2.69 | 1.59 | 2.25 | 1.89 |
| ***wbbK*** | 2.54 | 1.15 | 1.92 | 1.68 |
| ***yebW*** | 1.60 | 4.11 | 1.32 | 0.92 |
