# Supplementary material for: Global analysis of translation termination in E. coli
Source: PLoS Genet. 2017 Mar 16;13(3):e1006676. doi: 10.1371/journal.pgen.1006676 (PMC5373646; doi:10.1371/journal.pgen.1006676)
Supplement: S5 Table — The distribution of stop codons of the 84 genes classified as likely or possible recoding events in S1 and S2 Tables is shown in the observed column. The expected values were calculated using the frequencies in which each codon, UAA, UAG and UGA occur across the E.coli K-12 genome. The Chi-squared value was calculated to be 26.277 with 2 degrees of freedom, which is statistically significant by two-tailed t-test with a p-value of less than 0.0001. (DOCX) [file pgen.1006676.s015.docx]

| Stop Codon | Observed | Expected |
| --- | --- | --- |
| UAA | 40.5% (n=34) | 64.3% (n=54) |
| UAG | 11.9% (n=10) | 7.1% (n=6) |
| UGA | 47.6% (n=40) | 28.6% (n=24) |
| Total | 100% (n=84) | 100% (n=84) |

Chi-squared = 26.277 df=2 p-value <0.0001
